# Genome-wide differential expression of genes and small RNAs in testis of two different porcine breeds and at two different ages

**DOI:** 10.1038/srep26852

**Published:** 2016-05-27

**Authors:** Yao Li, Jialian Li, Chengchi Fang, Liang Shi, Jiajian Tan, Yuanzhu Xiong, Bin Fan, Changchun Li

**Affiliations:** 1 Key Lab of Agriculture Animal Genetics, Breeding, and Reproduction of Ministry of Education, College of Animal Science and Technology, Huazhong Agricultural University, Wuhan, 430070, People’s Republic of China; 2 Guangxi Yangxiang Pig Gene Technology limited Company, Guigang, 537120, People’s Republic of China; 3 Guangxi Yangxiang Incorporated Company, Guigang, 537100, People’s Republic of China

## Abstract

Some documented evidences proved small RNAs (sRNA) and targeted genes are involved in mammalian testicular development and spermatogenesis. However, the detailed molecular regulation mechanisms of them remain largely unknown so far. In this study, we obtained a total of 10,716 mRNAs, 67 miRNAs and 16,953 piRNAs which were differentially expressed between LC and LW pig breeds or between the two sexual maturity stages. Of which, we identified 16 miRNAs and 28 targeted genes possibly related to spermatogenesis; 14 miRNA and 18 targeted genes probably associated with cell adhesion related testis development. We also annotated 579 piRNAs which could potentially regulate cell death, nucleosome organization and other basic biology process, which implied that those piRNAs might be involved in sexual maturation difference. The integrated network analysis results suggested that some differentially expressed genes were involved in spermatogenesis through the ECM–receptor interaction, focal adhesion, Wnt and PI3K–Akt signaling pathways, some particular miRNAs have the negative regulation roles and some special piRNAs have the positive and negative regulation roles in testicular development. Our data provide novel insights into the molecular expression and regulation similarities and diversities of spermatogenesis and testicular development in different pig breeds at different stages of sexual maturity.

Small RNAs (sRNA, 19–35 nucleotides in size) are derived from diverse genome contexts, which involve intergenic regions, transposable elements and their remnants, or originate from viruses^1^,^2^,^3^ and non-coding regulatory molecules with diverse functions in various pathways^4^,^5^. sRNAs play critical roles in direct mRNA degradation^6^, translational repression^7^, heterochromatin formation^8^ and DNA elimination^9^. Currently, at least three types of sRNAs have been described: microRNAs (miRNAs) which are generated from hairpin-shaped precursors^10^, small interfering RNAs (siRNAs) which originate from double-stranded RNAs (dsRNAs)^11^ and PIWI-interacting RNAs (piRNAs) which are expressed specifically and abundantly in spermatogenic cells^5^.

Recent studies have indicated that sRNAs including miRNA^12^,^13^,^14^ and piRNA^15^,^16^ play important roles in the regulation of gene expression related to spermatogenesis or/and testicular development. In detail, over-expression of miR-34c, in the later stages of spermatogenesis, can down regulate the *TGIF2* gene, a repressor of the TGF-β signaling pathway which is crucial for spermatogenesis^17^. MiR184, miR21 and miR449 are associated with the functional regulation of gonadal somatic cells; miRNAs play diverse roles in the development and physiology of gonadal cells in mammalian reproduction^18^. Mael129, a conserved piRNA pathway protein Maelstrom, is associated with piRNA precursors and exhibits the reduced translation of numerous spermatogenic mRNAs, including those encoding acrosome and flagellum proteins^19^.

Testicular development and spermatogenesis are the major processes in male reproduction^20^. Germ cells are the direct participants in spermatogenesis, which is the key in reproduction. Spermatogenesis is a complex process through which diploid germ cells proliferate and differentiate into haploid spermatozoa^21^. This complex process of cell differentiation is controlled by many factors and fundamentally orchestrated through the expression of thousands of protein-encoding genes, which are developmentally regulated during spermatogenesis and play pivotal roles during specific phases of germ cell development. Previous studies have identified 1,652 spermatogenesis-related genes in the developing testis; 351 of these genes are expressed only in the male germ cells, with germ cell-specific transcripts being much less common earlier in testicular development^22^. Some genes for normal sperm are also indispensable. For example, the absence of *ADAM2* can lead to abnormal functions of sperm^23^. *Tnp2* translation is necessary for sperm maturation and male fertility^24^. Inversely, *Max* represses stem cell development^25^. Numerous genes are more significantly expressed in meiotic and/or early haploid spermatogenic cells than in somatic cells but inefficiently translated^26^. Existing evidence suggests the fluctuating expression of genes at different testicular development stages and the differential expression of genes in various testicular cells. During the testicular development and spermatogenesis, well-ordered and sequential changes in gene expression in each cell type are required to create a fully functional testis which is capable of producing mature spermatozoa^27^, necessary for sexual maturation.

Lu Chuan (LC) boars derived from Guangxi Zhuang Autonomous Region in China are characterized by a small body size and early sexual maturity; conversely, Large White (LW) boars derived from Canada feature a large body size, rapid growth and late sexual maturity. LC and LW boars considerably differ in sexual maturation time; LW boars reach maturity at ~180 days old^28^, whereas LC boars reach maturity at 90 days old^29^. Spermatogenesis is an important factor determining sexual maturation time and process. Previous studies proved that miRNA^30^ or piRNA^31^ are present in pig testes at sexual immaturity and maturity. However, the similarities and diversities of genes and sRNA, as well as their regulatory relationship, in the spermatogenesis and testicular development of different pig breeds at different maturity stages remain unclear.

In the present study, Illumina GAIIx sequencing technology was employed to profile miRNAs, piRNAs and mRNAs in the testes of LC and LW boars at sexual immaturity and maturity to investigate differentially expressed miRNAs, piRNAs and genes related to spermatogenesis and testicular development. The results of this study indicate that specific sRNAs and genes play important regulatory roles in testicular maturation, and contribute to sexual maturation differences between pig breeds.

## Results

To obtain a comprehensive view of genes and sRNAs expressed in sexually immature and mature porcine testicular tissues, Illumina GAIIx deep sequencing technology was employed on mRNA and sRNA libraries prepared from the testes of three LC and LW boars aged 20 days (sexual immaturity, B) and 200 days (maturity, A). No genetic linkage was present between the three LC boars or three LW boars at 20 or 200 days old.

### Whole genome expression profiles of mRNA

For each sequencing library, raw reads reached more than 2.4 million, 67.91% (sexual immaturity, B) and 90.60% (maturity, A) clean reads remained after removing adaptor sequences and low quality contaminant reads (Supplementary Table S1). The library effective length was 69 nt; the 99% score exceeded Q28 from 1 nt to 67 nt. Approximately 80% of the reads could be mapped onto the Sus scrofa reference genome (sscrofa10.2); 87.60–90.84% of the reads were in single position alignments (Supplementary Table S2). In the transcriptome data, uniquely mapped reads were mostly from mature mRNAs; the abovementioned mapping results showed that most of the mature mRNAs were polyA RNAs or ncRNAs, whereas most of the multiple mapped reads corresponded to rRNAs and tRNAs. Thus, unique mapped reads could be used for subsequent statistical analysis to ensure the reliability of the analysis results. The total gene amount from 12 samples was 22,288; the expressed genes were 84.96% of all S. scrofa genes (Supplementary Table S3). Interestingly, the reads for each sample were mapped mainly in the CDS, intron region and intergenic region. The reads from immature testis were located primarily in the intergenic and intron regions, whereas the reads from mature testis were widely distributed in the CDS region (~90% increase compared with the read number from the immature group), intron region (slightly reduced), and intergenic region (~50% significantly reduced compared with the immature group; Supplementary Figure S1). The results suggest that a large number of unannotated transcripts in the intergenic region are involved in the testicular maturation. The reads in the intron region had a high proportion of distribution, indicating the occurrence of several intron retention events.

### Genome-wide statistical evaluation of sRNA

For each sRNA library, raw reads reached more than 2 million, and at least 55.10% (sexual immaturity, B) and 87.94% (maturity, A) clean reads remained (Supplementary Table S4). The 100% score exceeded Q38 from 1 nt to 28 nt in SE40, whereas the 87–99% score outstripped Q28 from 1 nt to 69 nt in SE80. The results showed that the library and sequencing quality were feasible. Interestingly, the sRNA length was mainly 21–22 nt in all mature testis samples. However, the sRNA length was predominantly 28 nt, only a few of which were 21–22 nt in all immature testis samples. A classic miRNA molecule is 21–22 nt, whereas a classic piRNA is 28 nt. Thus, we predominately obtained miRNAs from mature testes and piRNAs from immature testes. Furthermore, miRNAs and piRNAs might have different important functions in regulating testicular maturation. Among the 84.81–95.14% reads mapped onto S. scrofa reference genome, 69.70–82.10% were in single position alignments (uniquely mapped; Supplementary Table S5). In addition, ~60% reads concentrated in the non-coding exon region in immature testes and ~70% were in the intergenic region in mature testes for the two pig breeds (Supplementary Table S6). These results pointed out that many sRNA might exclusively participate in testicular maturation. These were similar to a previous report with 30 and 180 day-old pig testis samples^30^.

Clean reads of sRNA were aligned to the Rfam database and were classified on the basis of RNA type. Reads of immature testes were 52.24–91.08% and reads of mature testes were 17.14–49.29% (Supplementary Table S7). Approximately 86.26–97.27% reads of immature testes were classified in miRNA, 1.04–4.50% in tRNA and 0.85–3.97% in snRNA. On average, 48.80% (LW) and 71.47% (LC) reads of mature testes were classified in miRNA, 37.68% and 14.03% in tRNA and 5.30% and 4.04% in snRNA, respectively, with a small number in other RNAs (Supplementary Table S8). This finding coincided with the analysis results of the genome (Supplementary Tables S5–S6) and miRBase (Supplementary Tables S9–S10). The results indicate that the diversity of mature miRNAs in LC and LW boars is relatively abundant and that miRNA regulation in testicular maturation differs between early and late sexual maturity pigs.

Effective clean reads were aligned to mature miRNAs of miRBase. From 10.24% (immature) to 63.20% (mature) of the total reads could be mapped to mature miRNAs (Supplementary Table S9). Interestingly, the ratio of mature miRNA reads of immature testes was significantly higher than that of mature testes in LW boars by two-tailed T test (P = 0.00108). However, the difference was not significant in LC pigs (P = 0.212), which may result from the significantly earlier sexual maturity of LC pigs than LW pigs. This finding agrees with previous results. Reads that were not mapped to mature miRNAs were aligned to the precursor miRNA (pre-miRNA) of miRBase. From 0.48% (mature) to 64.61% (immature) of the clean reads were derived from the non-mature pre-miRNA (Supplementary Table S10). These findings were similar to the alignment results of mature miRNA. The proportion of pre-miRNA reads was higher in immature testes than in mature testes in LW and LC pigs. These results suggest that miRNAs are involved in the sexual maturation and testicular development^32^.

### Differential expression analyses of genes

**Figure 1:**
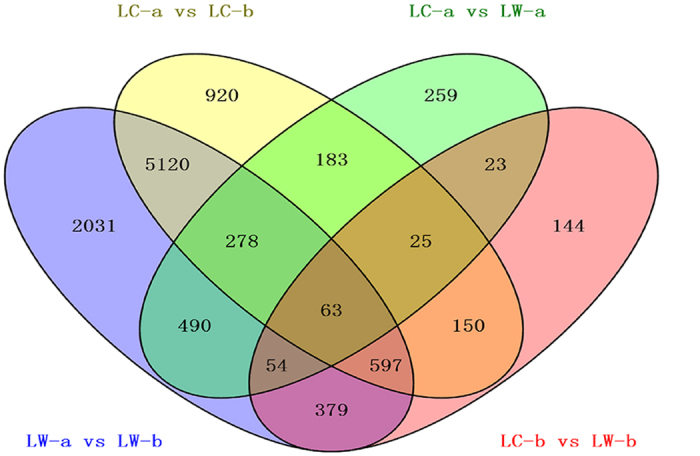
Complex of within-breed and inter-breed comparison for differentially expressed genes. A total of 10,716 unique DEGs (up-regulated and down-regulated genes), 2,645 DEGs are in inter-breed and 10,290 DEGs are in within-breed.

Correlation analysis of the gene expression showed that the correlation coefficient was relatively low (0.65–0.79, Supplementary Figure S2) between paired samples suggesting a presence of several differentially expressed genes (DEGs) between paired samples. The cluster results of gene expression between paired samples (Supplementary Figure S3) showed that the immature and mature testicular tissue samples from both LC and LW pigs gathered together. Gene expression differences were more remarkable in testicular maturation time points than that in different pig breeds. Three replicated individuals of LC or LW pigs did not initially cluster together at immature and mature stages of testicular development, indicating the presence of some intrinsic differences between individuals within breeds. A total of 10,716 DGEs among 22,288 expressed genes existed between the mature and immature testes of LW and LC pigs. However, the difference was much smaller at the same testicular maturation period (Table 1). Of the 10,716 unique DEGs, 63 co-existed in the within-breed and inter-breed comparisons (Fig. 1), which may be crucial in the process of sexual maturity. These genes include *KIT*, which regulates the development of testicular germ cell tumours^33^, and *ROBO2*, a downstream gene in the JAK–STAT signaling pathway, which is an important maintenance pathway for both germ line and cyst stem cells in the testis^34^.

### Gene ontology and pathway analyses of DEGs

We conducted gene ontology and pathway analyses using the DEGs mentioned in Table 1 to assess whether or not the genes were differential in some biological processes or pathways.

**Figure 2:**
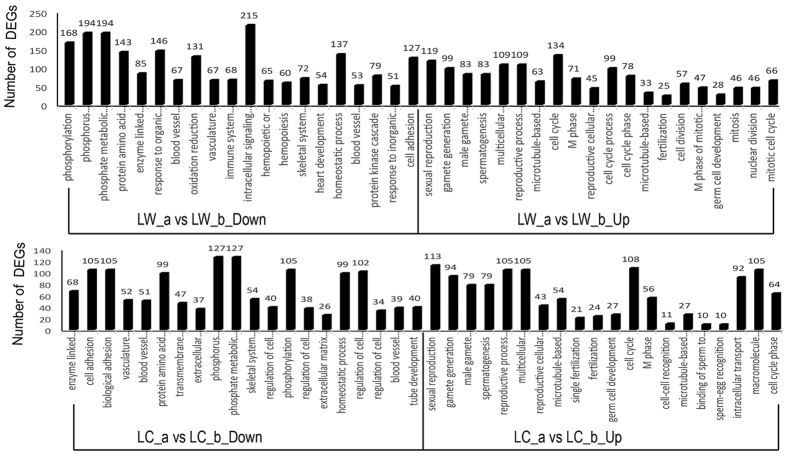
GO term of within-breed comparison of DEGs. The Top 20 GO (biological process) term analyses of DEGs of LW_a vs LW_b and LC_a vs LC_b.

For within-breed comparison (Fig. 2; Supplementary Table S11) in LW, 4,266 highly expressed genes in immature testes were assigned to 545 GO BP terms and 24 pathways (P < 0.05), including cell adhesion, blood vessel development, regulation of cell migration, stimulation of endogenous response and TGF-β signaling pathway; by contrast, 4,746 highly expressed genes in mature testes were assigned to 208 GO BP terms and 14 pathways, including maturity of sperm, cell cycle, germ cell development, spermatogenesis, fertilization, mitosis, meiosis (**not shown in** Fig. 2) and mTOR signaling pathway. In LC pigs, 3,138 highly expressed genes in immature testes enriched in 424 GO BP terms and 18 pathways involved in cell adhesion, regulation of cell migration, regulation of cell development, gonadal development and TGF-β signaling pathway. Meanwhile, 4,198 DEGs in mature testes were assigned to 165 GO BP terms and 13 pathways involved in spermatogenesis, cell cycle, germ cell development, fertilization and mTOR signaling pathway. These results suggest that the mature testis samples from LC pigs but not those from LW pigs were completely mature and that testicular development-related genes were differentially expressed between sexual maturity time points and pig breeds.

Functional clustering analysis of DEGs between LC and LW pigs in the same sexual maturity periods was conducted for inter-breed comparison (Supplementary Figure S4; Supplementary Table S12). Before sexual maturity, the up-regulated DEGs were preferentially enriched in sexual reproduction, meiotic cell cycle and spermatogenesis, whereas the down-regulated DEGs were related to cell adhesion, biological adhesion and the PPAR signaling pathway. After sexual maturity, the up-regulated DEGs were preferentially involved in response to oxidative stress and the PPAR signaling pathway, whereas the down-regulated DEGs were concerned with the regulation of secretion and developmental maturation. In the early stage of testicular development, the expression levels of genes related to sexual reproduction and spermatogenesis (*MAEL, SYCP1, PIWIL1, PIWIL2* and *DMC1*) were significantly higher in LC pigs than in LW pigs, indicating that the sexual development of LC pigs was earlier than that of LW pigs.

### Differential expression analyses of miRNA

The correlation coefficients between different samples were above 0.8, indicating a high relativity between samples (Supplementary Figure S5). For both mature miRNAs and pre-miRNAs, the number of TPM > 10 (Supplementary Table S13) was slightly lower in immature testes than that in mature testes. This result indicates that the regulation of sexual maturation is considerably complicated. In LC and LW pig testes, 297 miRNAs were detected, including 67 DE miRNAs in within-breed and 4 in inter-breed comparisons (Supplementary Table S14).

**Figure 3:**
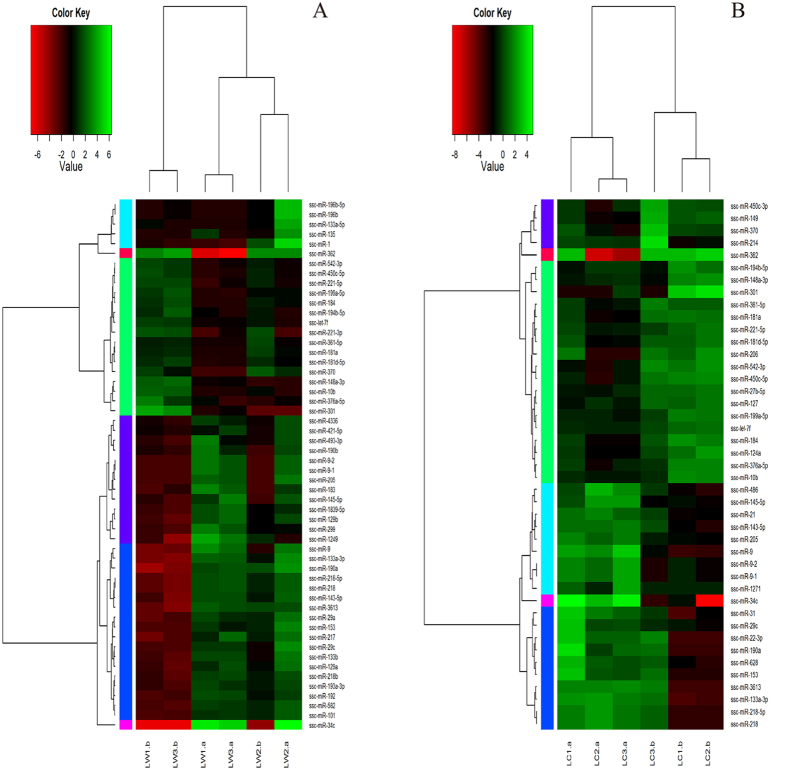
The expression of miRNAs in LW and LC pigs. (**A**) 54 DE miRNAs in LW pigs: 37 up-regulated expressed miRNAs and 17 down-regulated expressed. (**B**) 43 DE miRNAs in LC pigs: 20 up-regulated expressed miRNAs and 23 down-regulated expressed. Among these DE miRNAs, 14 were co-upexpressed and 16, meanwhile 16 were co-downexpressed.

In within-breed comparison, before and after sexual maturity, the number of DE miRNAs was 54 in LW pigs and 43 in LC pigs (Fig. 3; Supplementary Table S14). The number of significantly up-regulated expressed miRNAs was 37 in LW pigs, and 20 in LC pigs. Among these DE miRNAs, 14 were co-expressed, including miR-218-5p and miR-34c. The number of miRNAs with significantly down-regulated expression was 17 in LW pigs, and 23 in LC pigs, meanwhile 16 were co-expressed, including miR-184 and miR-221-5p. In inter-breed comparison, 2 DE miRNAs (miR-1249 and miR-196a) in LW pigs were up-regulated compared with LC pigs after sexual maturity, whereas only miR-31 was down-regulated. Before sexual maturity, only one DE miRNA (i.e. miR-34c) was down-regulated (Supplementary Table S14).

After building mature miRNA and pre-miRNA libraries, the effective sequencing reads of each sample were aligned to the libraries, and the expression of mature miRNA was calculated, mapped and annotated to predict the miRNAs. Novel miRNAs are predicted miRNAs with no homology to the miRbase (Supplementary Table S15). A total of 94 new miRNAs (TPM > 0) were discovered, 33 were co-expressed in LW and LC pigs, 69 in immature testes, 81 in mature testes, 81 in LW pigs and 75 in LC pigs (Supplementary Figure S6). The sequences and secondary structures of the predicted novel miRNAs were shown in Supplementary Table S16.

### Differential expression and function analyses of piRNA

In addition, 77,925 DE reads were obtained from the sRNA clean reads, and candidate piRNAs (sRNAs ≥ 26 nt) were predicted by a k-mer scheme with an online piRNA predicator (http://122.228.158.106/piRNA/analysis.php). We identified 16,953 DE piRNAs. All of these piRNAs were aligned against the gene region of the pig genome (Sscrofa10.2). Only sequences that perfectly matched the pig genome along their entire length were considered for further analysis. Taking both within-breed and inter-breed comparisons into account, we obtained 579 gene-derived piRNAs which originated from 305 unique genes (target gene) (Table 2). In addition, five exon-derived piRNAs originated from 12 genes. Gene-derived piRNAs constituted 3.42% of all piRNA categories. The minority (0.86%) of gene-derived piRNAs was mapped to exons of mRNAs, which strongly suggested that the gene-derived piRNAs were generated from primary transcripts. More piRNAs were up-regulated at maturity; the within-breed differences were more significant than the inter-breed differences. The results indicate that piRNAs are also involved in sexual maturation^35^.

Function analyses were performed on the original genes with DAVID for within-breed and inter-breed comparisons to investigate further the functions of DEG-derived piRNAs. In within-breed comparison, 200 and 20 days were compared, and only one biological process (regulation of oxidoreductase activity) involved genes of down-regulated piRNA in LC pigs. Inversely, genes of up-regulated piRNA were enriched with GO BP terms liking sulfur metabolic process, induction of apoptosis, induction of programmed cell death, regulation of transcription, DNA-dependent, regulation of RNA metabolic process, positive regulation of apoptosis, protein oligomerisation, positive regulation of programmed cell death and nucleosome organization in LC pigs; by contrast, the terms nucleosome assembly, chromatin assembly or disassembly, protein–DNA complex assembly, DNA packaging and sulfur metabolic process were identified in LW pigs (Supplementary Table S17), which are mainly involved in the regulation of cell survival and gene expression. Pathway analysis (Supplementary Table S18) showed that piRNA-generating genes played important roles in three pathways, namely, RNA degradation, renal cell carcinoma, and systemic lupus erythematosus (SLE), all of which were associated with gene expression. None of the GO BP terms or pathways in the inter-breed comparison resulted from the minority of gene-derived piRNAs and target genes.

### Integrated function analysis of gene and miRNA expression

MiRNAs can be incompletely complementary base paired to the target mRNA to inhibit its expression. The true DE target genes (TDETGs) came from the results that the target genes of DE miRNAs and the DEGs overlapped (Supplementary Table S19).

In within-breed comparison, 3,641 TDETGs were in LW pigs (54 DE miRNAs) and 2,843 TDETGs in LC pigs (43 DE miRNAs). In inter-breed comparison, 92 TDETGs were observed after sexual maturity (3 DE miRNAs) and 37 TDETGs before sexual maturity (1 DE miRNA). Subsequently, we conducted the within-breed comparison to find the key TDETGs related to spermatogenesis. Biological function analysis (GO analysis) results (Supplementary Table S20) for these TDETGs in LW pigs after sexual maturity were compared with those before sexual maturity. Results showed that the down-regulated genes were enriched in cell adhesion, bioadhesion, stimulation of endogenous responses, hormone responses, steroid hormones to stimulate the response, cell motility and cell migration-related biological processes, whereas the up-regulated genes were mainly enriched in nuclear fission, mitosis, cell division, sexual reproduction, spermatogenesis and gamete production. In addition, the down-regulated genes in LC pigs were mainly enriched in cell adhesion, bioadhesion, cell motility and cell migration, whereas the up-regulated genes were mainly enriched in nuclear fission, mitosis, cell division, sexual reproduction, spermatogenesis and gamete production. The main functions of TDETGs were similar between LW and LC pigs. Pathways (both in LC and LW) for the TDETGs (Supplementary Table S21) within the breeds before sexual maturity involved highly expressed genes related to cell development and gender differentiation signaling pathways, including ECM–receptor interaction, TGF-β, PPAR and focal adhesion. After sexual maturity, TDETGs between the breeds were related to growth and reproduction signaling liking RNA degradation, endocytosis and oocyte meiosis. The abovementioned results suggest that slight differences exist between pig breeds and large differences exist between different time points of sexual maturity in LC and LW pigs.

In the inter-breed comparison, only the GO BP terms were significant (Supplementary Table S22). When LW pigs were compared with LC pigs after sexual maturity, 71 down-regulated TDETGs mainly concerned the adhesion and regulation of hormone levels; 21 up-regulated TDETGs were enriched in gonadal development, reproductive structure development and development of primary sexual characteristics. Before sexual maturity, only one GO BP term (‘response to oxidative stress’) was identified from the up-regulated TDETGs.

### Network analysis of TDETGs

Spermatogenesis is subject to various factors; several miRNAs and their target genes are involved in the regulation of this complex process. The analytical results of miRNAs and mRNAs using the Spermatogenesis Online database (http://mcg.ustc.edu.cn/sdap1/spermgenes/prediction.php) and the ReCGiP database (http://klab.sjtu.edu.cn/MDpigs/), as well as the clustering results of DAVID, were combined; 30 co-expressed DE miRNAs and 46 TDETGs closely related spermatogenesis were screened (Table 3).

**Figure 4:**
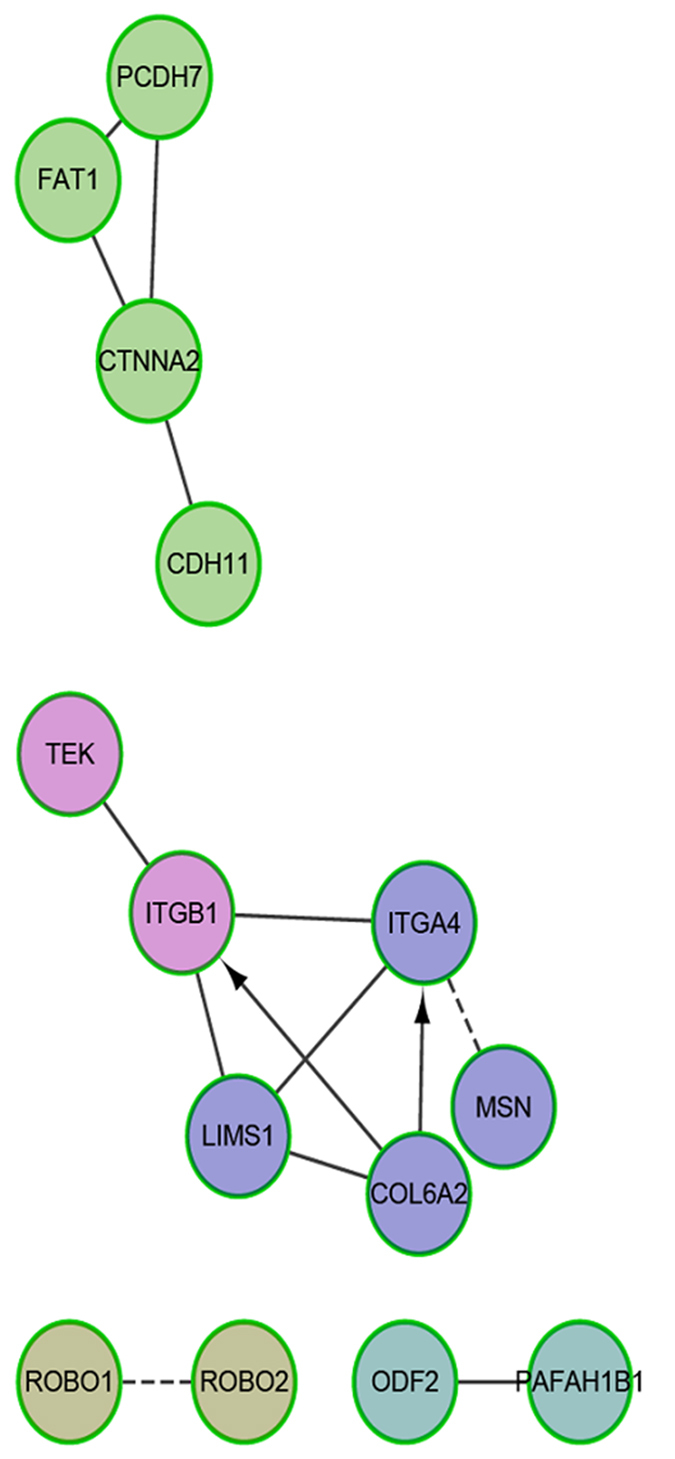
Concise functional interaction network of selected TDETGs. The effect of the interaction is represented by arrows, straight line and imaginary line. “− >” for activating/catalyzing, “−” for FIs extracted from complexes or inputs, and “−” for predicted FIs.

In consideration of certain important biological processes and pathways, the functional interaction (FI) network of TDETGs was used to identify major functional genes. The FI network of TDETGs was constructed for within-breed and inter-breed comparisons, but few differences were found between breeds (Supplementary Figures S7a–c). Many TDETGs, such as *ITGB1, ITGA6, MAPK6, SDC1, COL6A2, SRC, EGR1* and *NFYA,* were found to be key node genes enriched in the integrin signaling pathways, focal adhesion, ECM–receptor interaction and PI3K–Akt signaling pathway in within each breed. A concise FI network was constructed with the 46 selected TDETGs in the within-breed analysis, with *CTNNA2, ITGB1, ITGA4, LIMS1* and *COL6A2* as the central node genes (Fig. 4). The central node genes were involved in the ECM–receptor interaction, Wnt signaling pathway, PI3K–Akt signaling pathway and focal adhesion.

### Regulatory miRNA–mRNA–piRNA network

sRNAs are riboregulators that play critical roles in post-transcriptional regulation in most eukaryotes. In consideration of the differential expression levels of sRNAs and mRNAs, a regulatory network was constructed for miRNA–mRNA–piRNA (miRNAs, mRNAs, piRNAs were differentially expressed; DE mRNAs were the predicted target genes of DE miRNAs and the derived genes of DE piRNAs). The control mechanism of piRNAs remains unclear; thus, the results were integrated with the positive and negative regulation of piRNAs and the negative regulation of miRNAs.

**Figure 5:**
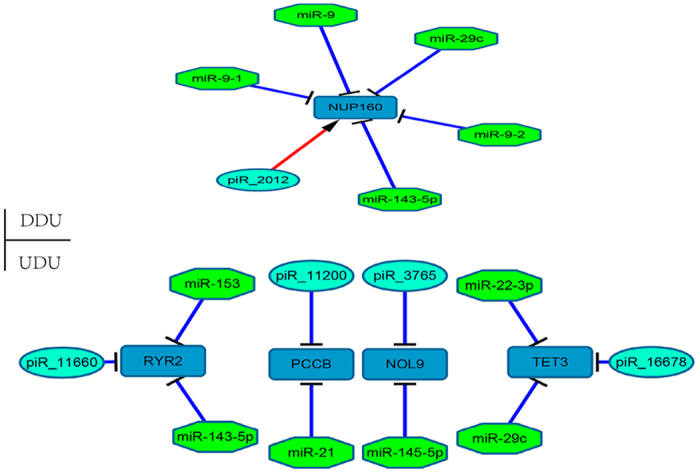
Integrated network of miRNA–mRNA–piRNA. Comparison between before and after sexual maturity in within-breed. DDU: down-regulated piRNA and down-regulated mRNA and up-regulated miRNA; UDU: up-regulated piRNA and down-regulated mRNA and up-regulated miRNA, “− >” for activating, “−” for inhibition.

A comparison within breeds between before and after sexual maturity, revealed four types of integration: I, up-regulated piRNAs, down-regulated mRNAs and up-regulated miRNAs (UDU); II, down-regulated piRNAs, down-regulated mRNAs and up-regulated miRNAs (DDU); III, up-regulated piRNAs, up-regulated mRNAs and down-regulated miRNAs (UUD); IV, down-regulated piRNAs, up-regulated mRNAs and down-regulated miRNAs (DUD). Two types (UDU and DDU) in LC pigs, and three types (UDU, DDU and UUD) in LW pigs were noted (Supplementary Figures S8a–b), with more complex regulation in LW pigs than in LC pigs. A common integrated network of miRNA–mRNA–piRNA was obtained by combing LC and LW pig data (Fig. 5). A total of 5 miRNAs, 1 gene and 1 piRNA were in DDU, whereas 6 miRNAs, 4 genes and 4 piRNAs were in UDU. We found that miR-143-5p, miR-29c, miR-9, miR-9-1, miR-9-2 and piR_20121 were correlated with the target *NUP160*, whereas miR-22-3p, miR-29c and piR_16678 were correlated with the target *TET3*. These results indicate that the miRNA–mRNA–piRNA networks indirectly reflect the expression patterns of miRNAs, node mRNAS and piRNAs during testicular development and that understanding the structure of the miRNA/mRNA/piRNA network could provide more insight into similarities and difference of different pig breeds at different stages of sexual maturity. No intersection of miRNA–mRNA–piRNA was noted in the inter-breed analysis.

### Validation of DE miRNAs and TDETGs by Q-PCR

**Figure 6:**
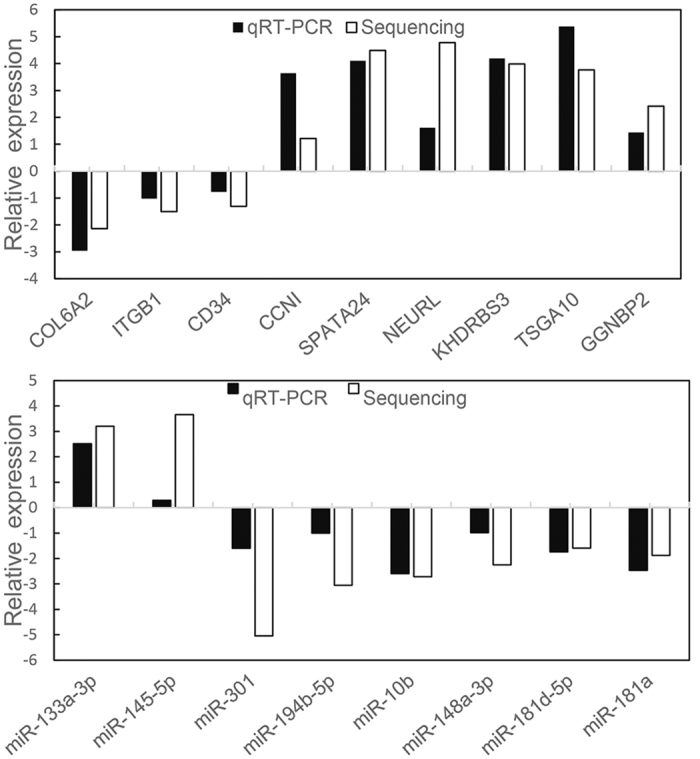
Validation of DE miRNAs and TDETGs by Q-PCR. Histograms of the relative expression levels of a/b (a: 200 days mature testes; b: 20 days immature testes) from LW and LC pigs. The x-axis represents the DE miRNAs and TDETGs, and the y-axis is the fold-change between the two groups (log(−ΔΔCt values, 2) for Q-PCR, log(a/b, 2) for sequencing). In total, the QPCR results have consisted with RNA-Seq results, three TDETGs (*COL6A2, ITGB1* and *CD34*) and four DE miRNAs (miR-10b, miR-148a-3p, miR-181d-5p, miR-181a) were down-regulated; four TDETGs (*SPATA24, KHDBPS3, TSGA10, GGNBP*) and one DE miRNA (miR-133a-3p) were up-regulated by two methods. Although the relative expressions of two genes (*CCNI* and *NEURL*) and three miRNAs (miR-145-5p, miR-301 and miR-194b-5p) have different patterns using Q-PCR and RNA-Seq, the direction of expression is same by two methods.

Quantitative real-time PCR was used to validate the nine TDETGs and eight DE miRNAs related to spermatogenesis and testicular development in LW and LC pigs as shown in Fig. 6. Q-PCR results were basically consistent with the sequencing data. Although the relative expression of two genes (*CCNI* and *NEURL*) and three miRNAs (miR-145-5p, miR-194b-5p and miR-301) differed using two approaches, the direction of expression is consistent by two methods, which was probably caused by biological differences between samples as well as the sensitivity and capability of the different methods. This trend indicates that our sequencing data are reliable, although the fold change may be slightly different.

## Discussion

In livestock production, the improvement of the reproduction performance of pigs is crucial, especially in boars. 20 and 200 days represent the young testis and sexual maturity stages, respectively. The key players in testicular development are Leydig, Sertoli and germ cells^36^. Leydig cells, the primary source of testosterone, can stimulate testicular development and promote spermatogenesis and maturation. Sertoli cells are specialised ‘nurse cells’ that provide the nutritional and architectural support required for germ cell development and secrete the androgen binding protein which joins the three cell types together. However, the molecular mechanisms underlying the sexual organ development of different pig breeds at different sexual maturity stages remain incompletely understood, especially from the point of view of sRNA and mRNA integrated analysis. In the present study, we constructed genome-wide expression profiles of sRNAs and mRNAs in a Chinese indigenous pig breed (LC) and a commercial pig breed (LW), conducted an integrated analysis and provided candidate key pathways and/or genes related to boar spermatogenesis and sexual maturity.

Most of the clean reads (mRNA: 79.05–85.50%, sRNA: 84.81–95.14%) identified in this study could match the released S. *scrofa* genome, which is similar to that described in the Chinese indigenous pig^37^. Based on the analysis of the sequencing data from the porcine testes of LC and LW pigs in two stages, a total of 22,288 genes, 297 miRNAs and 1,707,363 predicted piRNAs were obtained along with 10,716 DGEs, 67 DE miRNAs and 16,953 DE piRNAs; these results differed from those of previous studies^31^,^37^. This difference is probably related to the large span of the two time points of sampling (20 and 200 days-old) for the pig breeds (LC and LW) employed in our study. Furthermore, no genetic relationship between LC or LW boars was observed at 20 or 200 days old. The number of clean reads of mRNAs and sRNAs from the sexual maturity group significantly increased 31.83% and 30.04% compared with the sexually immature group, respectively. These molecular differences might lead to the phenotypic differences related to the spermatogenesis and sexual organ development of LC and LW pigs before and after sexual maturity.

Testicular development and spermatogenesis are intricate biological processes which are regulated by different genes and biological pathways at the genetic and epigenetic levels in the different developmental stages. This concept has been well represented in the biological comparison between LC and LW pigs. Based on the basic profiles of the genes, the differences between the two stages (immature and mature) were notable, although some cross points still existed. A total of 10,716 unique DEGs, 9,012 DEGs in LC pigs and 7,336 DEGs in LW pigs were detected after comparison before and after sexual maturity.

Interestingly, some DEGs are involved in spermatogenesis and testicular development. For example, within the breeds, *TGFBR1, BMPR2, SMAD2* and so on, which are members of the transforming growth factor-β (TGF-β) superfamily of proteins, were highly expressed in the immature testis samples. Their gene products play vital roles during testicular development and before spermatogenesis via the TGF-β signal pathway^38^. These genes also regulate the presence of the follicle stimulating hormone (FSH), which plays a key role in immature testicular development and can influence germ cell and Sertoli cell development and ultimately fertility^39^. Therefore, these DEGs probably play important regulatory roles in the early development of porcine testis. However, some up-regulated genes are vital in sexual maturation. For example, *AKT1* gene played a vital function in spermatogenesis, sperm maturation, and fertilization^40^. Abnormal expression of *AKT1* can directly affect spermatogenesis and sexual maturity, thereby resulting in male infertility^41^. *EIF4E* and *EIF4E2,* which are members of translational factor eukaryotic initiation factor 4E, stimulate translation with the poly(A)-binding protein partner Paip2a during late spermiogenesis in mice^42^. In mature testes, *AKT1, EIF4E* and *EIF4E2* are highly expressed to enrich in the mTOR signaling pathway, which confirms the results of a previous study^43^. It is probably that the mTOR signaling pathway participates in the proliferation and stimulation of meiotic initiation of spermatogonial stem cells (SSCs), thereby inducing the differentiation of preleptotene spermatocytes.

Difference of LC and LW pigs were observed in immature and mature testes. The expression levels of genes were significantly higher in LC pigs than that in LW pigs before puberty (*MAEL, SYCP1*) and sexual maturity development (*PIWIL1 and PIWIL2*). The *MAEL* gene, which plays an important role in sperm maturation, is composed of complexes with piRNA and Piwi proteins during meiosis to control methylation^44^. *SYCP1* (synaptonemal complex protein 1), causes Sertoli cells to secrete autocrine, paracrine and endocrine signals for early spermatogonial expression throughout the testis^45^. *PIWIL1* and *PIWIL2* (the PIWI subfamily gene) are specifically expressed in the testis and preferentially present high expression levels in the gonads of adult male pigs during development. In particular, *PIWIL1* is significantly increased in the testes of sexually mature males^46^, which may imply that the LW boars (200 days old) investigated in this study wouldn’t have fully attained sexual maturity for the continuous sperm production since the age of sexual maturity in LW boars ranges (including 200 days). These key genes that control sexual maturation are expressed earlier or at higher levels in LC pigs than in LW pigs, which possibly explain earlier sexual maturity of LC pigs than LW pigs.

Much documented evidence has proved that epigenetic modifications, including DNA methylation, RNA editing and small RNA/miRNA regulation participate in spermatogenesis and testicular development^47^. In addition, piRNAs regulate male germ cell meiosis and repress transposons in spermatogenesis after meiosis^48^.

To date, 280 pre-miRNAs and 326 mature miRNAs were identified in the pig miRNA Base20.0 database. A total of 297 miRNAs in LC and LW pigs were detected in the present study. Differential expression of miRNAs were nearly consistent within a breed whether LC or LW at 20 days old or 200 days old. This result indicated that miRNAs played conserved regulating functions in spermatogenesis and testicular development in pigs. Recently, miRNAs have been associated with the functional regulation of gonadal somatic cells, namely, Leydig and Sertoli and germ cells in the testis; more studies on the role of miRNAs in testicular function have been expounded from male germ cells to spermiogenesis via a model of spermatogenesis^18^. Spermatogenesis is complex biological process undergone by any organism, making it susceptible to perturbations which result in male sterility.

In both pig breeds, 14 miRNAs were identically up-regulated after sexual maturity (Supplementary Table S14); the relative expression fold-change of miR-145-5p, miR-205, miR-34c, miR-9, miR-9-1 and miR-9-2 at 200 days old were more notable, especially that of miR-34c, which were consistent with the findings of Lian^30^ (Fig. 3). Previous studies showed that miR-34c alone could not induce the entire male reproductive process but still plays an important role in the differentiation of murine embryonic stem cells into male germ cells by targeting the retinoic acid receptor gamma gene (RARg)^49^ and in germ cells by being highly expressed in spermatocytes and round spermatids; miR-34c over-expression lead to down-regulation of the TGIF2 gene, an inhibitor of the TGF-β pathway which is crucial for spermatogenesis^17^. A total of 16 identical miRNAs were similarly down-regulated after sexual maturity (Supplementary Table S14). For example, miR-184 was majorly expressed in the brain and testes, and especially highly expressed during postnatal testicular development; miR-184 over-expression could promote the proliferation of a germ cell line^50^. miR-221 played a crucial role in maintaining the undifferentiated state of mammalian spermatogonia by repressing *KIT* expression^51^. These results possibly hint that the detected miRNAs play potential roles in spermatogenesis and testicular development.

Only the DE miR-21 showed a higher expression in LC mature testes than in LW mature testes within the breed. Previous studies found that miR-21 is important for cell cycle in the testis and ovary^52^,^53^. Moreover, miR-21 could be regulated by the transcription factor ETV5, which is critical for SSC self-renewal to maintain the SSC population^54^. In addition, in the inter-breed analysis, miR-34c showed a significantly lower expression in LW pigs than in LC pigs at 20 days. miR-196a also had a higher expression in LW pigs than in LC pigs at 200 days, which is associated with the zinc finger proteins and homeodomain proteins that are closely related to fetal testicular development and the subsequent adult fertility^55^. Various expression levels of these miRNAs in different pig breeds at the same stage might contribute to the differences in testicular development and sexual maturity of LW and LC pigs.

Considering that piRNAs are closely related to the development of germ cells by binding to the Piwi subfamily in 2006^56^, studies have been focused on Piwi proteins^57^, including the sequence annotation^58^ and prediction of known and novel piRNAs^59^ during spermatogenesis. The present study is the first to report the basic differential expression of piRNA in two pig breeds at two stages of sexual maturity. The number of DE piRNAs (within-breed: 16,869, inter-breed: 627) is much larger than that of DE miRNAs (within-breed: 67, inter-breed: 4). The within-breed and inter-breed differences in piRNA number and expression are larger and more complicated than those of miRNAs, which should be related to the mechanism of piRNAs that originate and function during spermatogenesis. miRNAs and piRNAs can silence gene transcription and regulate translation and mRNA stability^60^. Moreover, piRNAs can maintain germ line and stem cells by forming the PIWI-interacting RNA complex (piRC) with PIWI proteins which are essential for spermatogenesis^61^.

A total of 16,953 DE piRNAs were detected in the present study, which was significantly different with previous studies on human and porcine testes^31^,^62^. This result indicates that piRNA expression sharply fluctuates with time during testicular development across species. piRNAs are diversely distributed among exonic, intronic, intergenic and repeat sequences^63^. The piRNAs predicted in the present study were poorly enriched in genes; only 16,953 (3.42%) DE piRNAs were generated from genes, and 5 (0.86%) from exons, which are lower than the previous data in porcine populations^31^.

The Gene Ontology (GO) and Kyoto Encyclopedia of Genes and Genomes (KEGG) analyses of piRNA-generating genes showed that the functions were mainly enriched in terms of biological processes for the regulation of apoptosis and chromatin assembly or disassembly. The RNA degradation pathways at the maturity stage in LC or LW pigs in the within-breed comparison were mainly involved in transcriptional regulation and chromatin modification (Supplementary Table S17). In addition, several associated-disease pathways, including renal cell carcinoma and SLE, were extrusive (Supplementary Table S18). These results were similar to previous reports in porcine populations^31^. Interestingly, some histone genes that are exclusively expressed in mammalian testes at the pachytene stage of spermatocytes were simultaneously enriched in the nucleosome and chromatin assembly process, as well as in the SLE pathway; these genes include *HIST1H2BA, HIST2H3A, HIST1H2BD* and *HIST2H2BF*. Extensive changes in chromatin structure and histone gene H1t expression occur in spermatogenesis^64^. SLE is a multisystem autoimmune disease in patients during their reproductive years, with a remarkably low proportion in males^65^, and is accompanied by sperm abnormalities in male patients^66^. The high frequency of testicular Sertoli cell dysfunction in male SLE patients is associated with semen abnormalities^67^. A recent study has reported that piR-823 demonstrates *in vitro* and *in vivo* tumor suppressive activity in human gastric cancer cells^68^. Therefore, piRNAs regulate gene expression in fundamental functions, such as nucleosome and chromatin assembly, and are potentially involved in diseases by regulating disease-correlated mRNAs to affect sperm quality.

The differences in piRNA and derived genes at the same stage in the different pigs were subtle possibly because of the expression levels of piRNAs primarily involved in spermatogenesis and their fundamental functions. Compared with miRNAs, piRNAs have more specific and fundamental functions; piRNAs may have unidentified functions in testicular development, which need to be further addressed in the future.

Existing evidence implied that sRNAs participate in the molecular regulation of mammalian spermatogenesis and testicular development. Marcon *et al*.^69^ found that sRNAs are present in the nucleus, miRNAs are highly abundant in the meiotic prophase and located in the nucleolus of Sertoli cells and piRNAs are located in the meiotic nucleus.

In general, miRNAs play important regulatory roles in animal spermatogenesis and testicular development by targeting the mRNAs of protein-coding genes and repressing their post-transcriptional properties^70^. Consequently, miRNA up-regulation implies the down-regulation and decreased activity of the target genes. Thus, this phenomenon is an important step to identify the miRNA target genes and to understand their roles in gene regulatory networks. Integrated analysis of the expression profiles of miRNAs and mRNAs minimizes false positive rates and identifies the real target genes to achieve the abovementioned objectives^71^. The number of TDETGs was greatly concentrated by the overlap of DEGs and target genes of DE miRNAs; these TDETGs (Supplementary Tables S17a,b) were predominantly enriched in cell adhesion (sexual immaturity) and spermatogenesis (sexual maturity). The regulation of cell adhesion can control the sexual and asexual development^72^.

The signaling pathways stimulated by TDETGs play crucial roles in testicular development and spermatogenesis. In the present study, the DE pathways within different breeds (LW or LC) before sexual maturity signaling pathways involved in cell development and gender differentiation; these pathways include ECM–receptor interaction and focal adhesion, which are similar to the results of a previous study^73^. FSH and androgens could act on Sertoli cells in stage VIII to control the expression of miRNAs which operate in a coordinated manner to regulate cell adhesion pathways and male fertility^74^. Focal adhesion signaling transduction pathways activate the proliferation, differentiation and motility of Sertoli cells during early testicular development^75^. *COL6A2* (collagen, VI type, α2) plays a major role in cell adhesion and affects the secretion of testicular Leydig cells^76^. In the sequencing results, *COL6A2* (up-regulated expression at immaturity) might be regulated by miR-133a-3p. The Q-PCR results were basically consistent with the RNA-seq and sRNA-seq data. After sexual maturity, signaling pathways were related to growth and reproduction liking endocytosis. Endocytic pathway controls the amount of free hormones that enter cells through passive diffusion for sex hormone-binding globulin to bind to androgens and estrogens and play crucial roles in the development of reproductive organs^77^. These pathways regulate the endocytic trafficking and recycling of membrane components and several transmembrane receptors on Sertoli cells to perform a specific role in spermatogenesis^78^.

The number of TDETGs between breeds sharply decreased during the integrated analysis of the expression profiles of miRNAs and mRNAs without significant pathways among the results. Therefore, the mechanism of the miRNAs cooperating with the genes that affect the phenotypic differences between pigs is intricate.

In the concise FI network (Fig. 4), previous studies indicated that the pathways of central node genes participate in regulating testicular development and spermatogenesis. The Wnt/β-catenin pathway plays an important role in the suppression of mouse and human spermatogonia development^79^. The suppression of Wnt/β-catenin signaling is a prerequisite for the normal development of primordial germ cells^80^.

**Figure 7:**
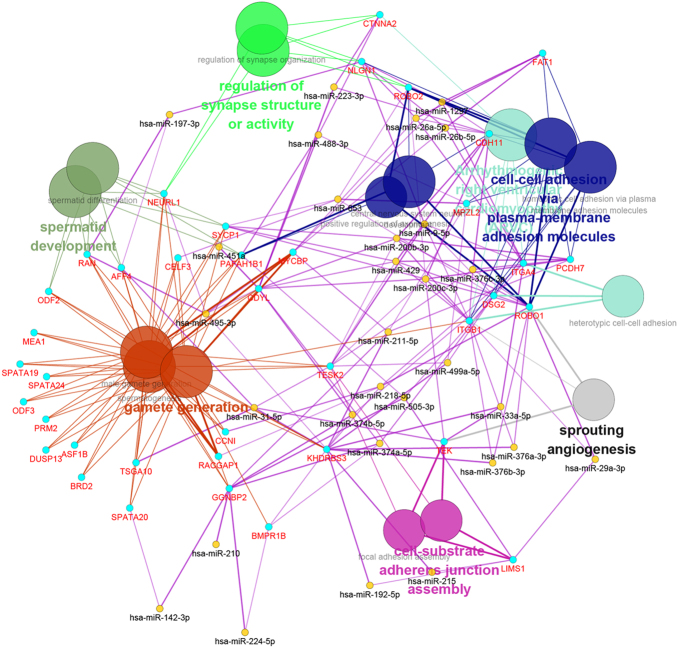
Integrated network analysis of RNA and miRNA. Analyses depend on within-breed comparison and results are in both LW and LC pigs. Different colors represent different biological processes, and similar biological processes are classified into one category, and seven major categories are exist.

An integrated regulation network was constructed to elucidate further the interaction between TDETGs and miRNAs (Fig. 7). Five central node genes (*CTNNA2, ITGB1, ITGA4, LIMS1* and *COL6A2*) and two miRNAs (miR218-5p and miR-221-5p) were detected in the integrated network. The biological processes (Supplementary Figure S9) covered seven major categories that are closely related to spermatogenesis (cell–cell adhesion via plasma membrane adhesion molecules, gamete generation, cell–substrate adherens junction assembly, arrhythmogenic right ventricular cardiomyopathy, regulation of synapse structure of activity, spermatid development and sprouting angiogenesis). Interestingly, we found that miR-221-5p targets the central node gene *ITGB1* and participates in gamete generation and cell–cell adhesion. *ITGB1* can also activate the PI3K–Akt signaling pathway^81^, which plays a core role in embryonic testicular cord formation^82^ and the self-renewing division of SSCs^83^. Finite TDETGs without especially significant pathways did not produce any of the node genes in the integrated regulation network (Supplementary Figures S7c and S10) in the inter-breed comparison.

In the miRNA-mRNA-piRNA regulatory network, two types (UDU and DDU) in LC pigs and three types (UDU, DDU and UUD) in LW pigs were detected. The numbers of miRNA, mRNA or piRNA sharply fluctuated in each type, especially piRNA in LW (UDU: 91, DDU: 2, UUD: 25). This trend ultimately led to the complexity of the integrated network and indirectly reflected the expression patterns in the testicular development in different stages and pig breeds. In the expression network, piR_2012 activates *NUP160* and piR_16678 represses *TET3*. These figures confirm the previous finding that piRNAs play multiple roles in maintaining the stability of mRNA^84^ and inhibiting gene expression^85^. *NUP160* functions in mRNA export^86^, *TET3* is involved in chromatin modification and can affect gonadal development^87^. miR-29c can exhibit a negative result with sperm DNA damage during spermatogenesis^88^; this miRNA negatively regulates *NUP160* and *TET3* in UDU and DDU. These networks of miRNA, mRNA or piRNA fundamentally regulate mRNA expression, which could highlight the importance of proper temporal regulation in the development of male germ cells or gonad.

## Conclusions

We obtained the genome-wide expression profiles of mRNAs, miRNAs and piRNAs and constructed an integrated molecular network of all three in mature and immature testes of LC and LW pigs. Within-breed DE molecules (LC or LW) at sexual maturity were much greater than the inter-breed DE molecules. Some mRNA, miRNA and/or piRNA genes and pathways were found to be common, which means they are likely to be essential for spermatogenesis and testicular development. And meanwhile, some mRNAs, miRNAs and piRNAs were found to be differentially expressed at 20 days old and 200 days old testes in these two breeds with different sexual mature ages, which means they may play different but important roles in testicular development and spermatogenesis. Our data provide new insights into the genetic similarities and diversities of testicular development and the associated process of spermatogenesis in distinct pig breeds. The detailed molecular mechanisms underlying the gene similarities and diversities of spermatogenesis and testicular development of the different pig breeds at different sexual mature ages require further studies.

## Methods

### Ethics statement

Animals care and all the experimentation in this study were carried out in accordance with the pre-approved guidelines from Regulation Proclamation No. 5 of the Standing Committee of Hubei People’s Congress. All experimental protocols were approved by the Ethics Committee of Huazhong Agricultural University, Wuhan City, Hubei Province, P. R. China.

### Sample collection and total RNA extraction

Three LC and LW boar pigs aged 20 days (sexually immature^89^) and 200 days (sexually mature^28^) were obtained from the Yang Xiang Pig Gene Technology Co., Ltd in the Guangxi Zhuang Autonomous Region of China. The pigs were castrated to obtain the testes samples. The testes samples were immediately snap-frozen in liquid nitrogen and then stored at −80 °C. Total RNA was extracted using Trizol reagent (Invitrogen, Carlsbad, CA, USA). RNA concentration and purity were detected with a NanoDrop 2000 Spectrophotometer (Thermo Scientific, USA). All the procedures were carried out according to manufacturer’s protocol.

### mRNA and sRNA library construction and sequencing

Total RNA was treated with RQ1 DNase (Promega, Madison, Wisconsin, USA) to remove DNA. For each sample, 10 μg of total RNA was used for RNA-seq library preparation. Polyadenylated mRNAs were purified and concentrated with oligo(dT)-conjugated magnetic beads (Invitrogen, Carlsbad, California, USA) before use for directional RNA-seq library preparation. Purified mRNAs were iron fragmented at 95 °C, followed by end repair and 5′ adaptor ligation. Reverse transcription was performed with the RT primers harboring the 3′ adaptor sequence and randomized hexamers. The cDNAs were purified and amplified. The PCR products corresponding to 200–500 bp were purified, quantified and then stored at −80 °C until used for sequencing. For high-throughput sequencing, single-end 80 bp sequencing of the cDNAs was performed on the Illumina GAIIx (Illumina, San Diego, CA, USA) by ABlife, Inc. (Wuhan, China).

Total RNA (3 μg) was used for sRNA cDNA library preparation with the Balancer NGS Library Preparation Kit for sRNA (GnomeGen, San Diego, CA, USA) in accordance with the manufacturer’s instructions. In brief, RNAs were sequentially ligated to 3′- and 5′-end adaptors, reverse transcribed to cDNA and then amplified by PCR. Whole libraries were applied to 10% native PAGE gel electrophoresis; bands corresponding to mRNA insertion were cut and eluted. After ethanol precipitation and washing, the purified sRNA libraries were quantified with a Qubit Fluorometer (Invitrogen, Carlsbad, California, USA) before cluster generation and two batches (40 and 80 nt) single-end sequencing analysis using the Illumina GAIIx (Illumina, San Diego, CA, USA) in accordance with the manufacturer’s instructions.

### Analysis of mRNA sequencing data

The obtained sequence reads (Fastq files) were checked by FastQC software. Raw sequence reads were processed into clean reads as follows: the two N-containing reads and the sequence adaptor were removed; low-quality reads were cut off, and the remaining reads with lengths 16 nt and above were considered clean. All clean reads were aligned to the pig reference genome (ftp://ftp.ncbi.nlm.nih.gov/genomes/Sus_scrofa/) by Tophat with 2 nt fault tolerance. The expression profile of mRNAs was reflected by the reads per kilobase of exon model per million mapped reads (RPKMs, RPKM = total exon reads/[mapped reads (millions) * exon length (kb)]. Correlation analysis of gene expression levels between samples were counted by corrgram package of R language. Cluster analysis of samples were performed to detect the degree of homogeneity in the samples used heatmap.2 of gplots package. The edgeR^90^ software package was applied to detect DEGs with P ≤ 0.01 and |log_2_ Ratio| ≥ 1. The functions of the DEGs were determined through the GO term and KEGG pathway annotation with the DAVID gene annotation tool^91^. Only GO-BP terms or KEGG pathways with P < 0.05 were considered significant.

### Analysis of sRNA sequencing data

After removing adapter sequences, low-quality reads and maintaining reads with lengths 14 nt and above, the clean reads were mapped to the whole pig genome by using Tophat to detect the reads distribution across pig genomic regions. The clean reads were matched to Rfam database (ftp://selab.janelia.org/pub/Rfam) to identify sequence tags originating from cisreg, lncRNA, miRNA, rRNA, sRNA, snRNA and tRNA. Unique sequences were aligned with miRBase 20.0 (http://www.mirbase.org/) to identify known porcine miRNAs (including mature and precursor miRNA). To determine new possible unknown miRNAs, samples were predicted by miRDeep2^92^. Candidate piRNAs were classified from sequences of 24–32 nt, which did not originate from known sRNAs, imperfectly mapped to the genome or transcriptome and aligned with piRNA database (http://pirnabank.ibab.ac.in/request.html). The expression profile of sRNA was normalized in transcripts per million (TPM) values (TPM = reads (sRNA) /total reads *10^6). Correlation and DE analyses of sRNA were similar to those of mRNAs. Target prediction and functional annotation of DE miRNAs were performed with Miranda^93^ and DAVID.

### RNA and sRNA analysis by synthesis

TDETGs were the overlap results that from the target genes of DE miRNAs and DEGs. The Reactome FI Plugin was used to visualise the FI network of TDETGs and to identify major functional genes with the Cytoscape software environment^94^. The integrated regulatory network was constructed using ClueGO^95^ and CluePedia^96^ to identify the functional regulations from the miRNAs to the mRNAs. On the basis of the differential expression of sRNAs and mRNAs, the regulatory network was constructed for miRNA–mRNA–piRNA interactions.

### Quantitative real-time PCR (Q-PCR)

Nine TDETGs (*CCNI, SPATA24, NEURL, KHDBPS3, TSGA10, GGNBP2, COL6A2, ITGB1* and *CD34*) *and* eight DE miRNAs (miR-301, miR-194b-5p, miR-10b, miR-148a-3p, miR-181d-5p, miR-181a, miR-133a-3p and miR-145-5p) from Table 3 were confirmed via real-time RT-PCR to be involved in spermatogenesis and testicular development. theFor each sample, the total RNA was incubated with RNase-free DNase Ι (Thermo, Massachusetts, USA) to remove DNA contamination. Subsequently, RNA was 3′-extended by a ploy(A) tail, and the miRNA was extended by stem-loop RT followed by qPCR^97^. First-strand cDNA was synthesized using the Thermo Scientific RevertAid First Strand cDNA Synthesis Kit (#1622; Fermentas, Lithuania). Finally, quantitative real-time PCR with gene-specific primers and miRNA-specific stem-loop RT primers (Supplementary Table S23) were performed on the Bio-Rad CFX384 Real-Time System with a SYBR Green Ι Real-time PCR Master Mix (QPK-201; Toyobo). All reactions were run in triplicate; porcine *RPL32* and *U6* snRNA were used as internal controls of mRNA and miRNA, respectively. Each reaction was performed in a 10 μL reaction mixture, containing 250 ng cDNA template, 5 μL the SYBR Green Real-time PCR Master Mix, 0.2 μM each primer pairs with the following PCR program: incubated at 95 °C for 2 min, followed by 40 cycles of 95 °C for 15 s, 60 °C for 15 s and 72 °C for 15 s. The difference in the expression level of genes or miRNAs between samples was calculated using the 2^−ΔΔCt^ method.

## Additional Information

**How to cite this article**: Li, Y. *et al*. Genome-wide differential expression of genes and small RNAs in testis of two different porcine breeds and at two different ages. *Sci. Rep.* **6**, 26852; doi: 10.1038/srep26852 (2016).

## Supplementary Material

Supplementary Information

Supplementary Information

Supplementary Information

Supplementary Information

Supplementary Information

Supplementary Information

Supplementary Information

Supplementary Information

Supplementary Information

## Figures and Tables

**Table 1:** Total number of differentially expressed genes (fold change ≥ 2 or ≤ 0.5, P < 0.01).

DEG sample	Total expressed genes	Up regulated gene NO.	Down regulated gene NO.	Total
Within-breed comparison				
LW-a vs LW-b	22288	4746	4266	9012
LC-a vs LC-b	22288	4198	3138	7336
Inter-breed comparison				
LC-a vs LW-a	22288	857	518	1375
LC-b vs LW-b	22288	928	507	1435

**Table 2:** Total number of DE piRNAs and target genes.

DE piRNA sample	Total piRNA	gene-derived piRNA	Target gene
Within-breed comparison			
LW-a down	252	6	16
LW-a up	13547	426	193
LC-a down	233	2	11
LC-a up	15874	513	243
Inter-breed comparison			
LW-a vs LC-a down	186	2	3
LW-a vs LC-a up	247	11	6
LW-b vs LC-b down	194	2	2
LW-b vs LC-b up	3	0	0

Gene-derived piRNAs which originated from unique genes (target gene).

**Table 3:** TDETGs involving spermatogenesis.

Lower expression of miRNAs after sexual maturation	Higher expression of target genes related to spermatogenesis	Lower expression of miRNAs before sexual maturation	Higher expression of target genes related to cell adhesion
ssc-miR-184	*LIG3*	ssc-miR-143-5p	*ITGB1, ROBO1*
ssc-miR-199a-5p	*RAN*	ssc-miR-153	*LIMS1, ROBO2*
ssc-miR-450c-5p	*MYCBP*	ssc-miR-205	*TEK, ROBO2, CDH11*
ssc-miR-148a-3p	*ODF2, KHDRBS3*	ssc-miR-133a-3p	*COL6A2, MSN, BMPR1B, CDH11*
ssc-miR-542-3p	*SPATA20, RAN, RACGAP1*	ssc-miR-29c	*ITGB1, ROBO1, FAT1, COL6A2*
ssc-miR-181d-5p	*CDYL, ODF3, RAN, TSGA10*	ssc-miR-34c	*LIMS1, DSG2, MSN, CDH11*
ssc-miR-361-5p	*MYCBP, PAFAH1B1, ODF2, PPP2R2B*	ssc-miR-3613	*LIMS1, OLR1, BMPR1B, CDH11*
ssc-let-7f	*MYCBP, ASF1B, AFF4, RACGAP1, CELF3*	ssc-miR-145-5p	*MPZL2, NLGN1, ITGA4, CTNNA2, CD34*
ssc-miR-181a	*CDYL, ODF3, RAN, SPATA18, GGNBP2*	ssc-miR-218	*NLGN1, CTNNA2, DSG2, ROBO1, ROBO2*
ssc-miR-362	*CDYL, MYCBP, ASF1B, AFF4, PRM2,*	ssc-miR-218-5p	*NLGN1, CTNNA2, DSG2, ROBO1, ROBO2*
ssc-miR-301	*CDYL, PPP2R2B, CCNI, RAN, CELF3*	ssc-miR-190a	*MPZL2, NLGN1, ITGA4, ITGB1, FAT1, ROBO2*
ssc-miR-376a-5p	*SYCP1, CDYL, MYCBP, PAFAH1B1, RAN, TESK2*	ssc-miR-9	*OLR1, PCDH7, CD34, ROBO1, MSN, CDH11*
ssc-miR-10b	*SPATA20, NEURL, MYCBP, ODF2, BRD2, MEA1, TESK2*	ssc-miR-9-1	*OLR1, NLGN1, ITGA4, PCDH7, CD34, ROBO1, TEK, MSN, CDH11*
ssc-miR-194b-5p	*MYCBP, ODF2, ASF1B, SPATA24, AFF4, TESK2, RACGAP1*	ssc-miR-9-2	*OLR1, NLGN1, ITGA4, PCDH7, CD34, ROBO1, TEK, MSN, CDH11*
ssc-miR-221-5p	*MYCBP, ODF2, LIG3, CELF3, TESK2, PRM2, PNMA1*		
ssc-miR-370	*CDYL, DUSP13, ODF3, ODF2, PPP2R2B, LIG3, ASF1B, RAN, CELF3, SPATA19*		

Biological process analysis for TDETGs which are selected when they participate in spermatogenesis and cell adhesion, and their opposite miRNAs.
