# Supplementary material for: Genome-wide differential expression of genes and small RNAs in testis of two different porcine breeds and at two different ages
Source: Sci Rep. 2016 May 27;6:26852. doi: 10.1038/srep26852 (PMC4882596; doi:10.1038/srep26852)
Supplement: Supplementary Information [file srep26852-s1.pdf]

Supplementary Information for:

Genome-wide differential expression of genes and small RNAs in testis of two different porcine breeds and at two different ages

Yao Li[1], Jialian Li[1,2], Chenchi Fang[1], Liang Shi[3], Jiajian Tan[3], Yuanzhu Xiong[1], Bin Fan[1,2], Changchun Li[1*]

[1]Key Lab of Agriculture Animal Genetics, Breeding, and Reproduction of Ministry of Education, College of Animal Science and Technology, Huazhong Agricultural University, Wuhan, 430070, People's Republic of China; [2]Guangxi Yangxiang Pig Gene Technology limited Company, Guigang, 537120, People's Republic of China; [3]Guangxi Yangxiang Incorporated Company, Guigang, 537100, People's Republic of China.

*the corresponding author (email: lichangchun@mail.hzau.edu.cn)

Supplementary files note: supplementary Figures S1-10 and supplementary Tables S1-10, S13-15, S19, S23.

The following supplementary material is available:

Supplementary Figure S1. RNA mapping distribution.

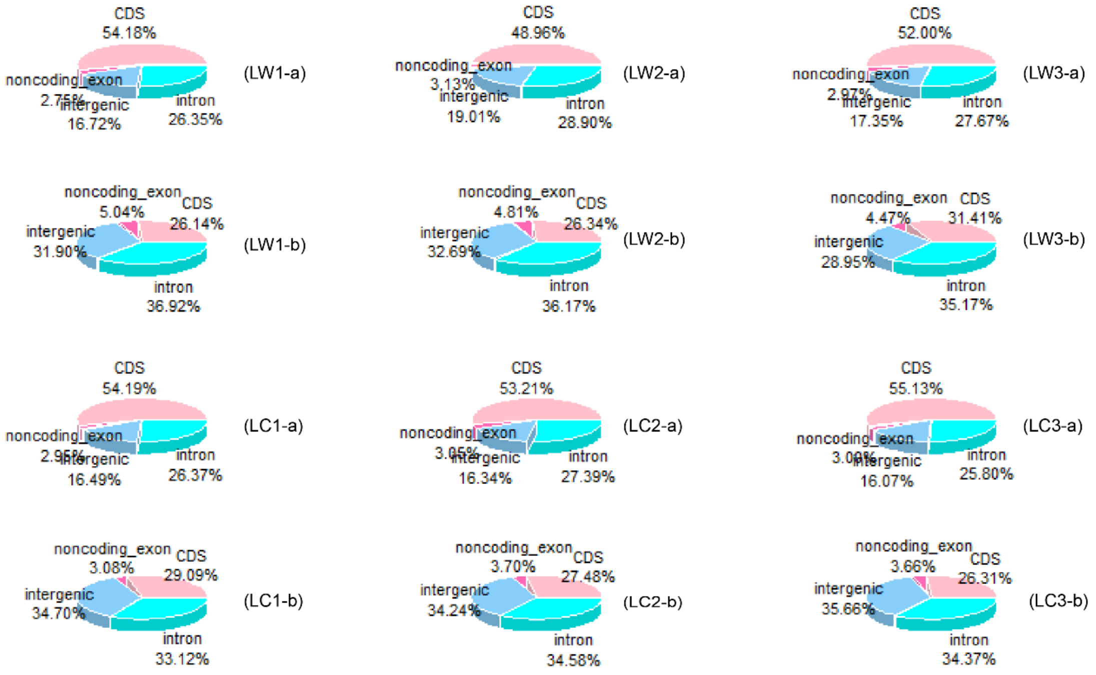

The first row: LW1-a, LW2-a, LW3-a; the second row: LW1-b, LW2-b, LW3-b; the third row: LC1-a, LC1-a, LC1-a; the last row: LC1-b, LC1-b, LC1-b. (LW: Large White , LC: Lu Chuan; a: mature testis, b: immature testis)

Supplementary Figure S2. Correlation analyses of the gene expression between paired samples.

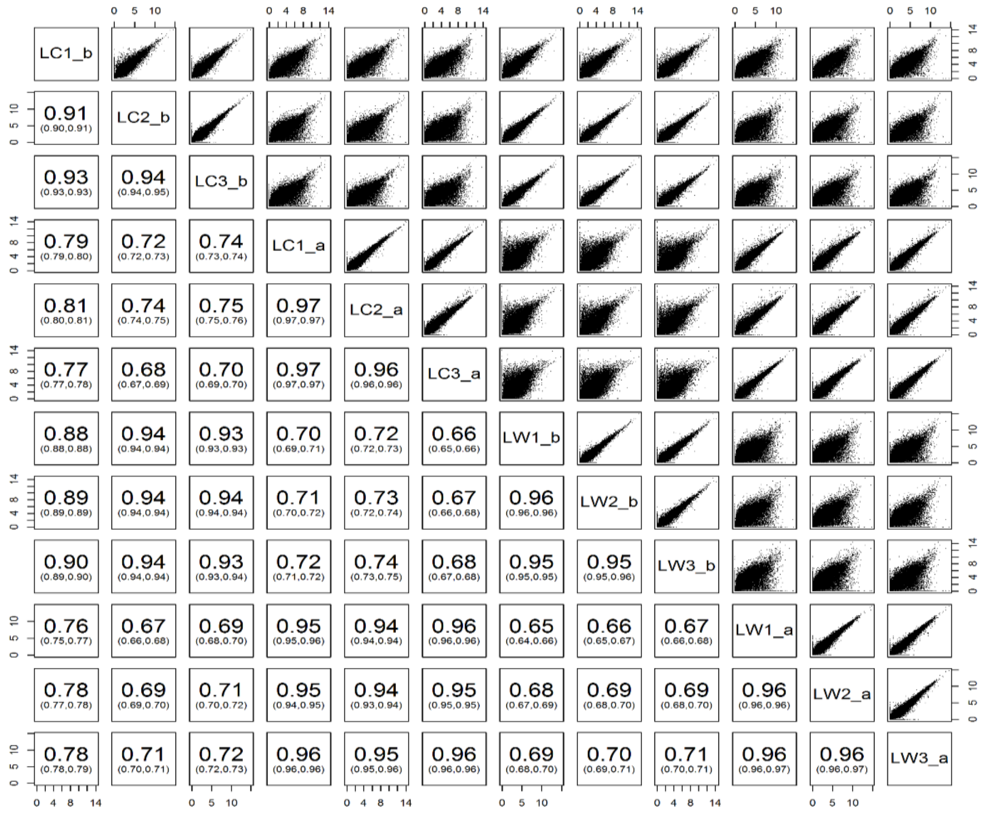

Supplementary Figure S3. Cluster analysis of gene expression of samples.

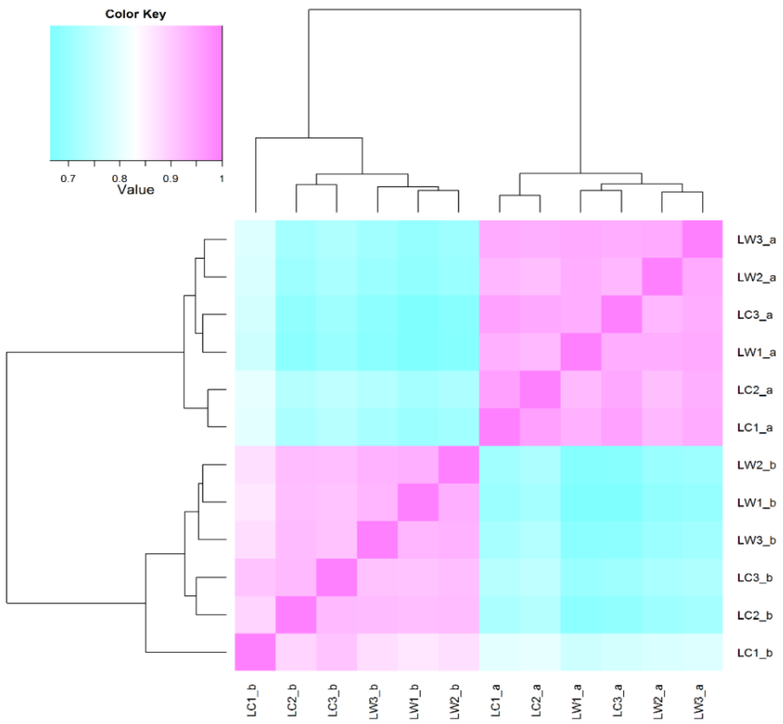

Immature and mature testicular tissue samples gather together, respectively, but the uniformity is poorer in mature testes (a) than immature testes (b), which can been seen in LC3-a and LW1-a.

Supplementary Figure S4. GO term of inter-breed comparison of DEGs.

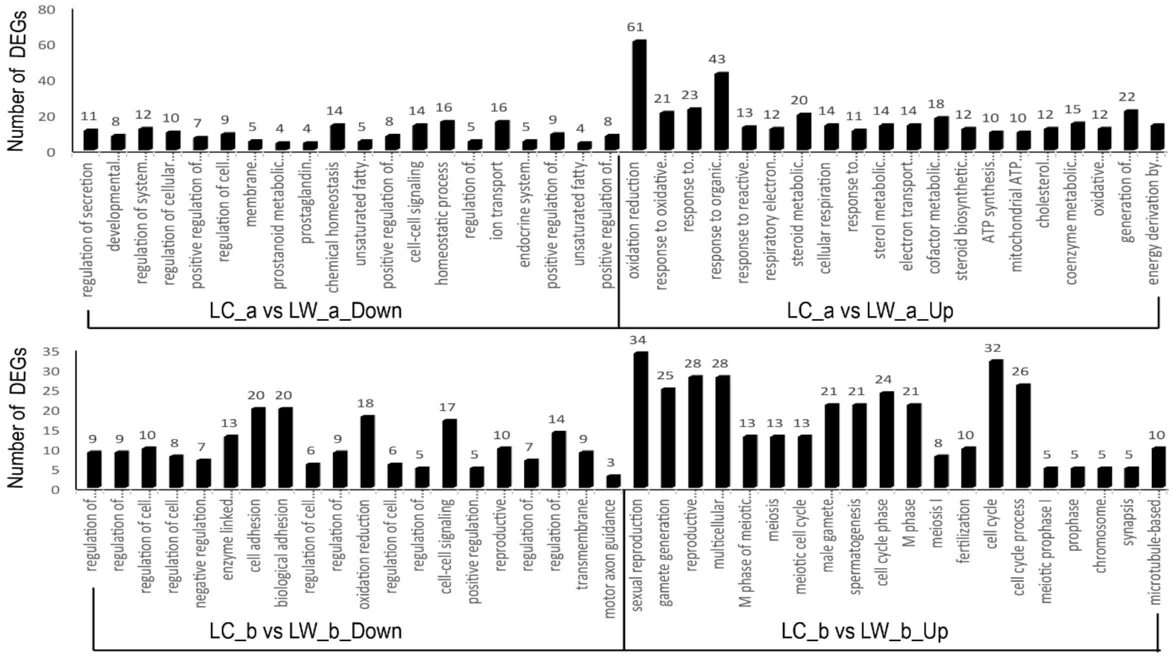

The Top 20 GO (biological process) Term analyses of DEGs of LC_a vs LW_a and LC_b vs LW_b.

Supplementary Figure S5. Correlation analyses of the miRNA expression between paired samples.

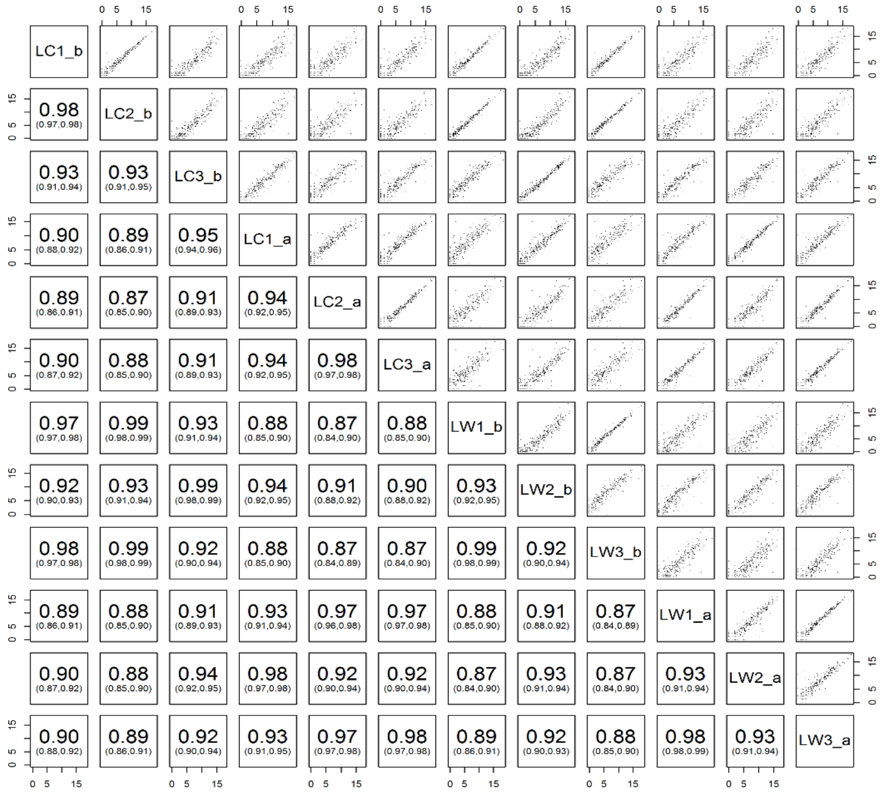

Supplementary Figure S6. Novel miRNAs in mature (a) and immature (b) testes.

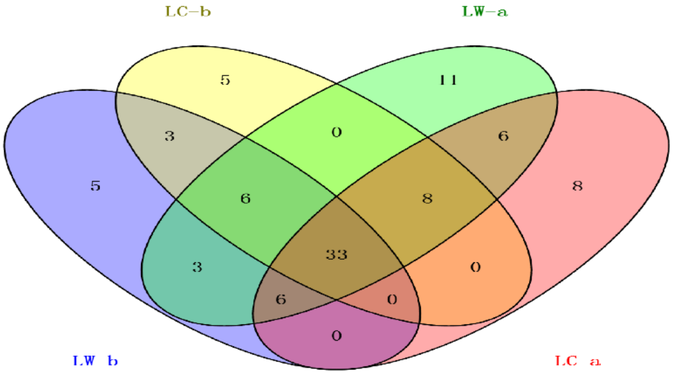

Supplementary Figure S7a. FI network of TDETGs in LW pigs in within-breed comparisons.

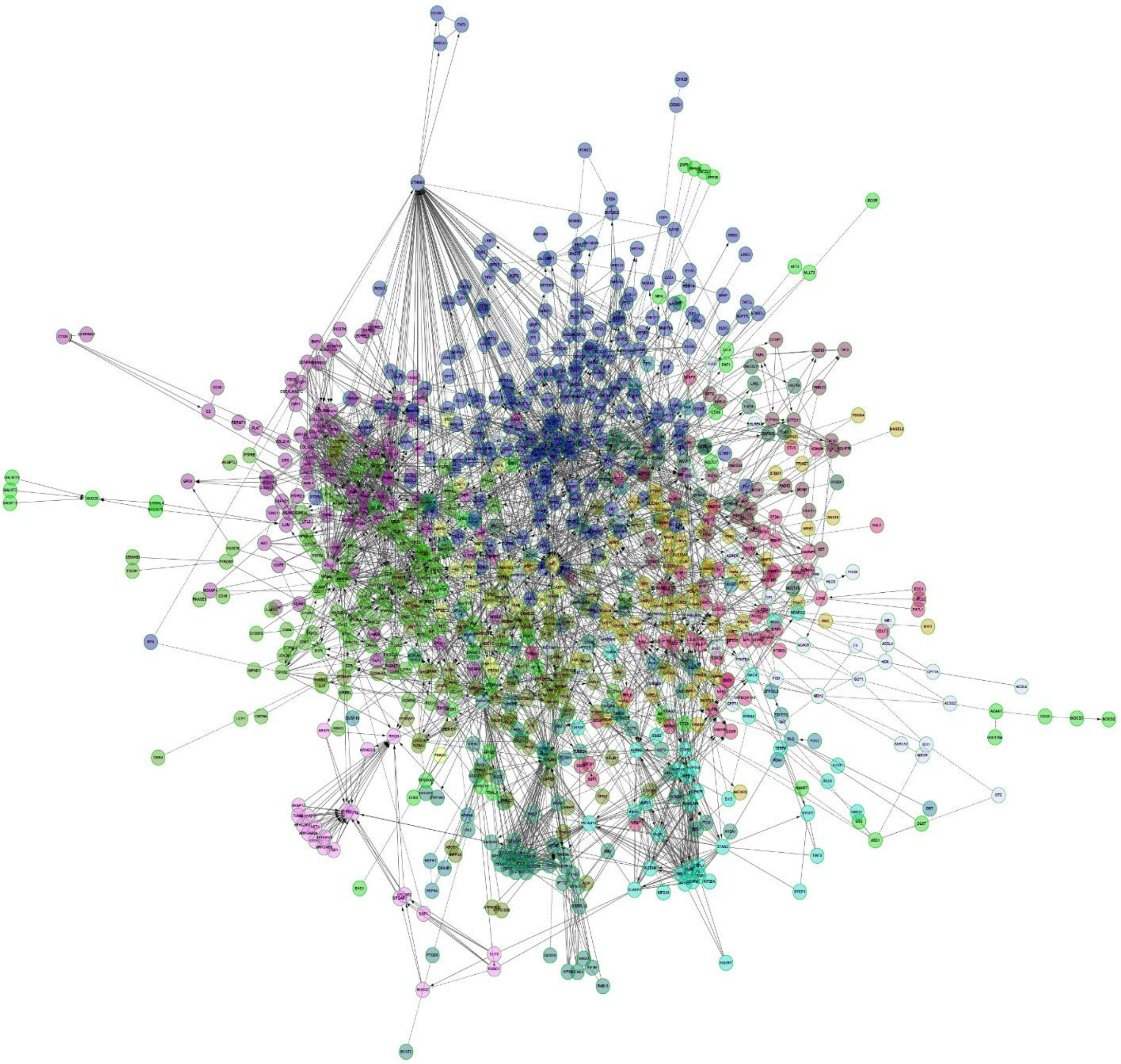

The effect of the interaction is represented by arrows, bar-headed lines, straight line and imaginary line. "->" for activating/catalyzing, "-|" for inhibition, "-" for FIs extracted from complexes or inputs, and "---" for predicted FIs.

Supplementary Figure S7b. FI network of TDETGs in LC pigs in within-breed comparisons.

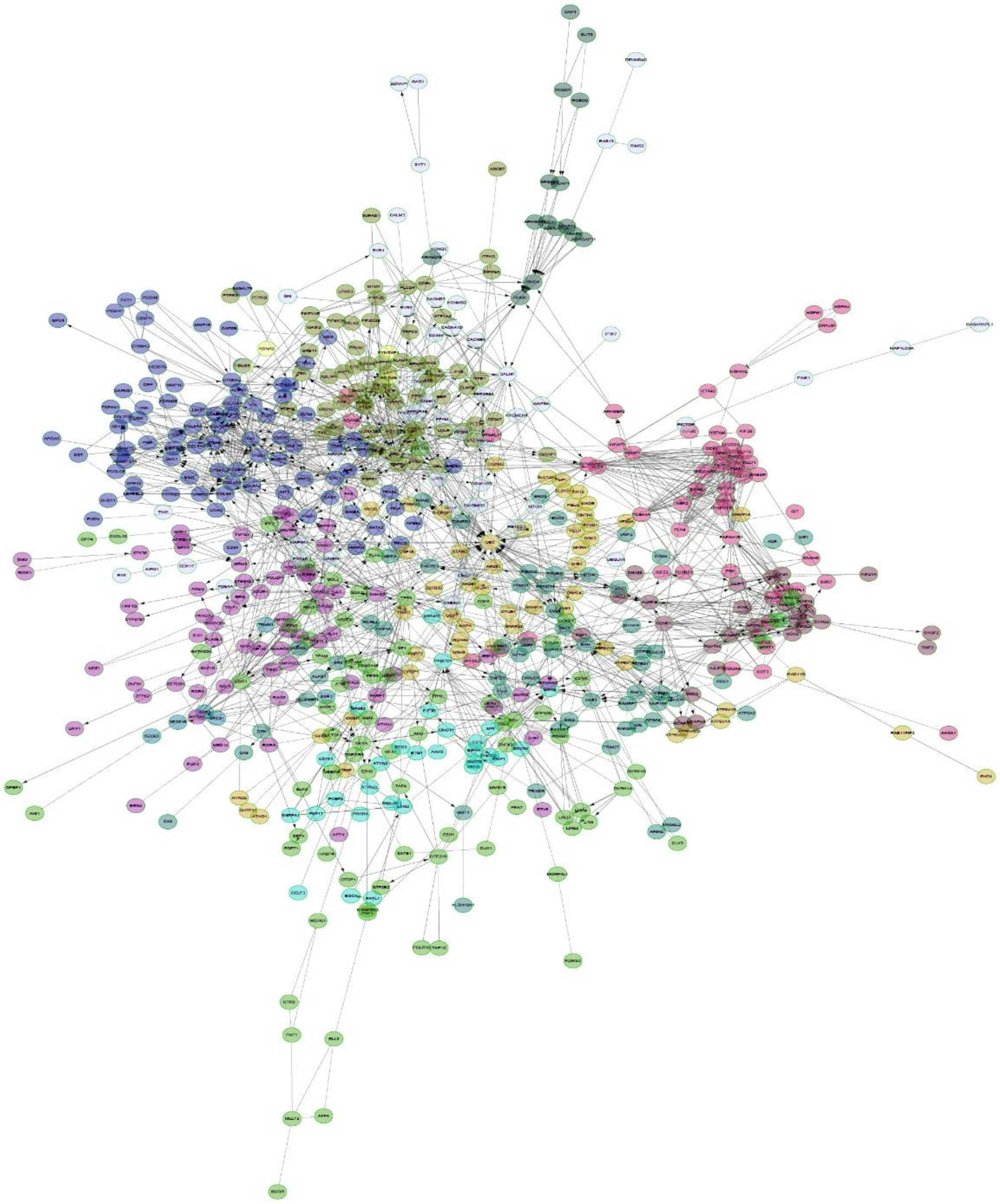

Supplementary Figure S7c. FI network of TDETGs in inter-breed comparison at maturity stage.

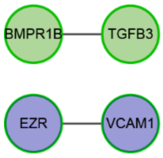

Supplementary Figure S8a. miRNA–mRNA–piRNA integrated network in LC pigs.

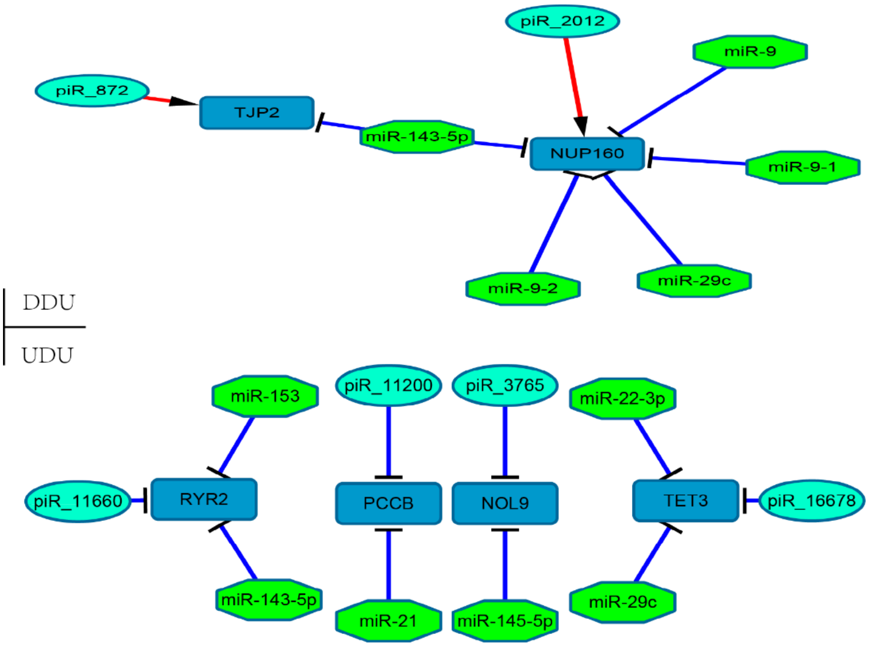

Comparison between after and before sexual maturity in inner-breed. DDU: down-regulated piRNA and down-regulated mRNA and up-regulated miRNA, UDU: up-regulated piRNA and down-regulated mRNA and up-regulated miRNA. "->" for activating, "-|" for inhibition.

Supplementary Figure S8b. miRNA–mRNA–piRNA integrated network in LW pigs.

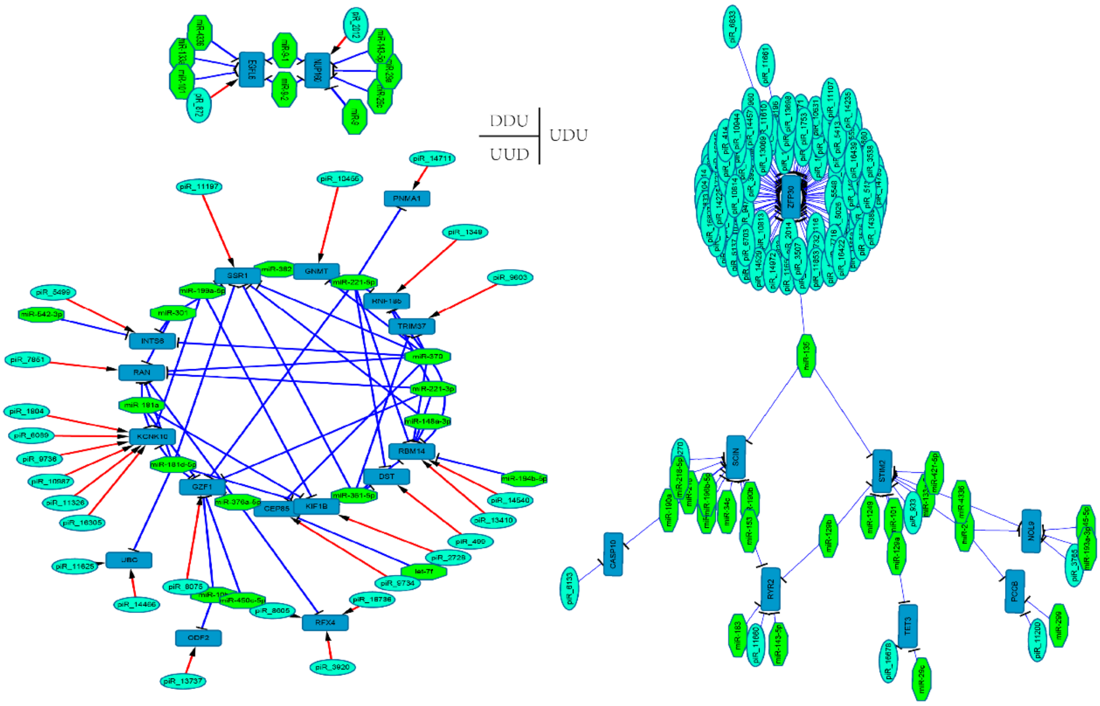

Comparison between after and before sexual maturity in inner-breed. DDU: down-regulated piRNA and

down-regulated mRNA and up-regulated miRNA, UDU: up-regulated piRNA and down-regulated mRNA and up-regulated miRNA, UUD: up-regulated piRNA and up-regulated mRNA and down-regulated miRNA. "->" for activating, "-|" for inhibition.

Supplementary Figure S9. Biological process of integrated network of RNA and miRNA. Analyses depend on inner-breed comparison, and results are in both LW and LC pigs.

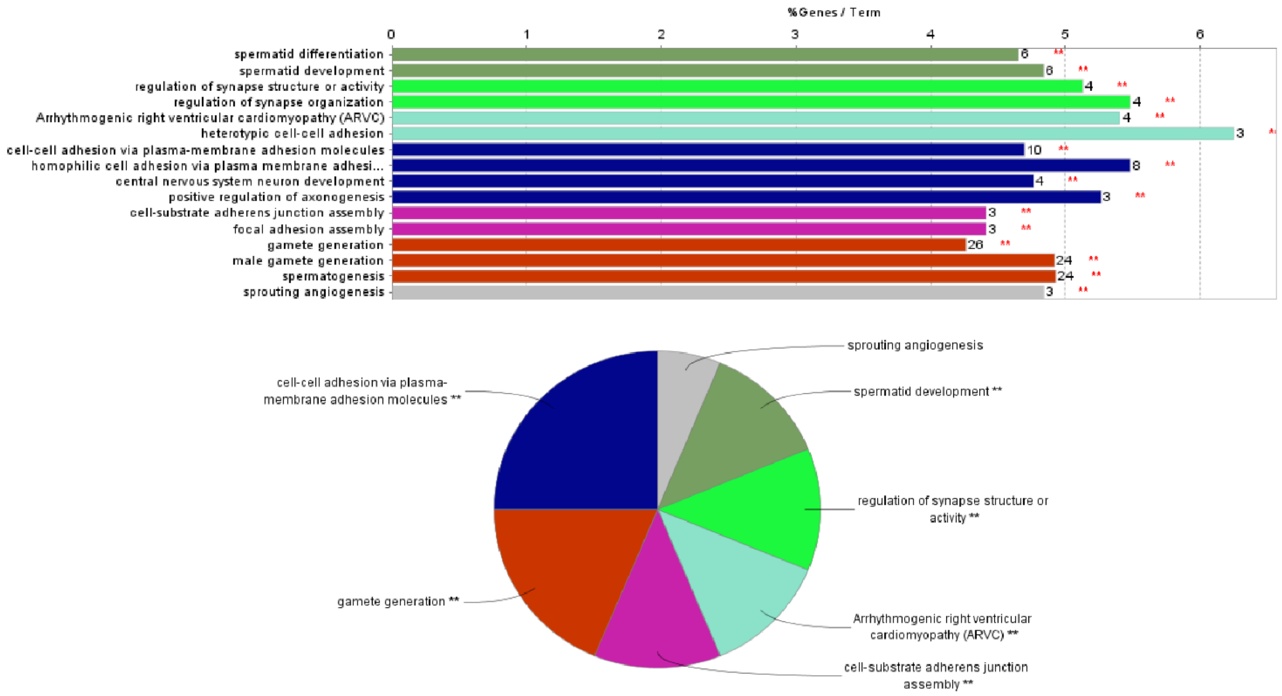

Supplementary Figure S10. Integrated network analysis of RNA and miRNA in inter-breed.

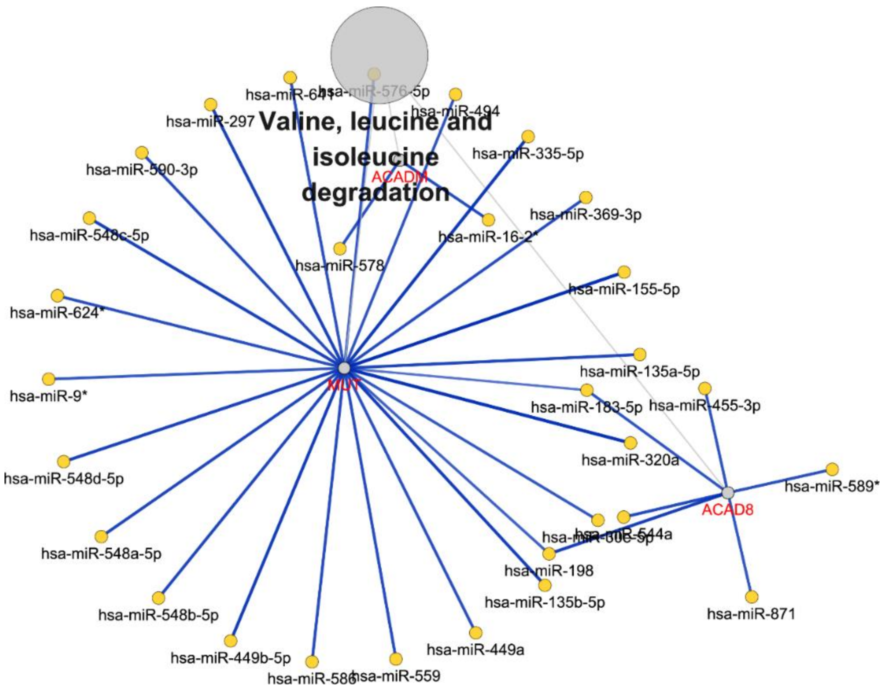

The results only represent the network in inter-breed comparison at maturity.

Supplementary Table S1. Obtain the high-quality RNA clean reads

Sample	Raw data	Clean reads
LW1-a	2438162	2246841(92.15%)
LW2-a	2448939	2218752(90.60%)
LW3-a	3367069	3089913(91.77%)
LC1-a	4512122	4159869(92.19%)
LC2-a	2837099	2615279(92.18%)
LC3-a	4332083	3989819(92.10%)
LW1-b	3411905	2317171(67.91%)
LW2-b	3395784	2363920(69.61%)
LW3-b	3179151	2224767(69.98%)
LC1-b	3077475	2159918(70.18%)
LC2-b	2498815	1728193(69.16%)
LC3-b	2770407	1970250(71.12%)

Supplementary Table S2. Mapping of clean RNA-seq reads on the Sus scrofa's genome

Sample	Clean reads	Total mapped	Unique mapped	Multiple mapped
LW1-a	2246841	1907758(84.91%)	1723410(90.34%)	184348(9.66%)
LW2-a	2218752	1835106(82.71%)	1650903(89.96%)	184203(10.04%)
LW3-a	3089913	2600459(84.16%)	2345938(90.21%)	254521(9.79%)

LC1-a	4159869	3556881(85.50%)	3219265(90.51%)	337616(9.49%)
LC2-a	2615279	2215327(84.71%)	2012503(90.84%)	202824(9.16%)
LC3-a	398919	3398838(85.19%)	3077310(90.54%)	321528(9.46%)
LW1-b	2317171	1857687(80.17%)	1633535(87.93%)	224152(12.07%)
LW2-b	2363920	1999660(84.59%)	1754792(87.75%)	244868(12.25%)
LW3-b	2224767	1878919(84.45%)	1658473(88.27%)	220446(11.73%)
LC1-b	2159918	1736211(80.38%)	1520925(87.60%)	215286(12.40%)
LC2-b	1728193	1378546(79.77%)	1220488(88.53%)	158058(11.47%)
LC3-b	1970250	1557523(79.05%)	1371784(88.07%)	185739(11.93%)

Total mapped% = Total mapped/ Clean reads; Total mapped: clean reads mapped on the Sus scrofa's genome;

Unique mapped% = Unique mapped/ Total mapped; Unique mapped: reads mapped only one region to the reference genome;

Multiple mapped% = Multiple mapped/ Total mapped; Multiple mapped: reads mapped more than one region to the reference genome.

Supplementary Table S3. Mapped reads on expressed genes

Sample	Total genes in Sus scrofa’s genome	Expressed genes (mapped reads number>0)	Expressed genes (mapped reads number>10)
LW1-a	26235	16845(64.21%)	9766(57.98%)
LW2-a	26235	16849(64.22%)	9671(57.40%)
LW3-a	26235	17658(67.31%)	11152(63.16%)
LC1-a	26235	18286(69.70%	12130(66.33%)
LC2-a	26235	17445(66.50%)	10837(62.12%)
LC3-a	26235	17980(68.53%)	11560(64.29%)
LW1-b	26235	17262(65.80%)	9690(56.13%)
LW2-b	26235	17587(67.04%)	10062(57.21%)
LW3-b	26235	17573(66.98%)	10116(57.57%)
LC1-b	26235	17771(67.74%)	10238(57.61%)
LC2-b	26235	17010(64.84%)	9114(53.58%)
LC3-b	26235	17272(65.84%)	9374(54.27%)

Expressed genes (mapped reads number> 0): This column is the number of expressed gene and the proportion of the total genes of pig genome;

Expressed genes (mapped reads number> 10): This column list the number of mapped reads number> 10 and the

proportion of mapped reads number> 10 to mapped reads number> 0

Supplementary Table S4. Obtain the high-quality sRNA clean reads

Sample	Raw data	Clean reads
LW1-a	9637482	8475007(87.94%)
LW2-a	6833191	6677570(97.72%)
LW3-a	6850400	6297075(91.92%)
LC1-a	11565119	11268970(97.44%)
LC2-a	2324114	2072267(89.16%)
LC3-a	5528729	5062403(91.57%)
LW1-b	5758978	3845999(66.78%)
LW2-b	6550138	5493010(83.86%)
LW3-b	5820998	3783088(64.99%)
LC1-b	6123056	4127967(67.42%)
LC2-b	6179004	3404493(55.10%)
LC3-b	4705610	4197678(89.21%)

Supplementary Table S5. Mapping of sRNA clean reads on the Sus scrofa's genome

Sample	Clean reads	Total mapped	Unique mapped	Multiple mapped
LW1-a	8475007	7891818(93.12%)	5678414(71.95%)	2213404(28.05%)
LW2-a	6677570	6205609(92.93%)	4536239(73.10%)	1669370(26.90%)
LW3-a	6297075	5757759(91.44%)	4465332(77.55%)	1292427(22.45%)
LC1-a	11268970	10488082(93.07%)	8666805(82.63%)	1821277(17.37%)
LC2-a	2072267	1971506(95.14%)	1551348(78.69%)	420158(21.31%)
LC3-a	5062403	4759252(94.01%)	3817784(80.22%)	941468(19.78%)
LW1-b	3845999	3458534(89.93%)	2790978(80.70%)	667556(19.30%)
LW2-b	5493010	5110836(93.04%)	3562361(69.70%)	1548475(30.30%)
LW3-b	3783088	3465021(91.59%)	2708148(78.16%)	756873(21.84%)
LC1-b	4127967	3621892(87.74%)	2973537(82.10%)	648355(17.90%)
LC2-b	3404493	2887427(84.81%)	2187424(75.76%)	700003(24.24%)
LC3-b	4197678	3939476(93.85%)	2810665(71.35%)	1128811(28.65%)

Total mapped% = Total mapped/ Clean reads;Total mapped: clean reads mapped on the Sus scrofa's genome;

Unique mapped% = Unique mapped/ Total mapped; Unique mapped: reads mapped only one region to the reference genome;

Multiple mapped% = Multiple mapped/ Total mapped; Multiple mapped: reads mapped more than one region to the reference genome.

Supplementary Table S6. sRNA-seq effective reads distribution across pig genomic regions

Sample	CDS	noncoding-exon	Intergenic	intron
LW1-a	79115.56(1.0%)	765341.65(9.7%)	5829496.26(73.9%)	1217864.53(15.4%)
LW2-a	63774.92(1.0%)	526424.38(8.5%)	4548728.52(73.3%)	1066681.17(17.2%)
LW3-a	53516.47(0.93%)	764395.24(13.28%)	4067832.36(70.65%)	872014.93(15.15%)
LC1-a	126414.64(1.2%)	853733.36(8.1%)	7693532.50(73.4%)	1814401.50(17.3%)
LC2-a	25479.88(1.3%)	632465.72(32.1%)	998725.74(50.7%)	314834.67(16.0%)
LC3-a	65364.23(1.4%)	569399.60(12.0%)	3344268.52(70.3%)	780219.65(16.4%)
LW1-b	11368.14(0.33%)	2466975.96(71.33%)	666956.60(19.28%)	313233.30(9.06%)
LW2-b	29344.23(0.57%)	2475252.15(48.43%)	1652698.78(32.34%)	953540.84(18.66%)
LW3-b	7541.16(0.22%)	2477529.07(71.50%)	646767.47(18.67%)	333183.30(9.62%)
LC1-b	62561.94(1.7%)	1479412.89(40.8%)	1546207.05(42.7%)	533710.11(14.7%)
LC2-b	11360.35(0.39%)	1786776.13(61.88%)	762317.02(26.40%)	326973.49(11.32%)
LC3-b	38049.31(0.97%)	1704060.20(43.26%)	1514074.16(38.43%)	683292.32(17.34%)

CDS%= CDS/ (CDS + noncoding-exon + Intergenic + intron);

noncoding-exon%= noncoding-exon/ (CDS + noncoding-exon + Intergenic + intron);

Intergenic%= Intergenic/ (CDS + noncoding-exon + Intergenic + intron);

intron%= intron/ (CDS + noncoding-exon + Intergenic + intron).

Supplementary Table S7. Proportion of sRNA clean reads mapped to Rfam

Sample	Clean reads	Total mapped	Unique mapped	Multiple mapped
LW1-a	8475007	2592420 (30.59%)	515609(19.89%)	2076811 (80.11%)
LW2-a	6677570	1785009(26.73%)	388573 (21.77%)	1396436 (78.23%)
LW3-a	6297075	1779278 (28.26%)	364553 (20.49%)	1414725 (79.51%)
LC1-a	11268970	1931982(17.14%)	361569 (18.71%)	1570413 (81.29%)
LC2-a	2072267	1021430 (49.29%)	89983 (8.81%)	931447 (91.19%)
LC3-a	5062403	1187075 (23.45%)	157495 (13.27%)	1029580 (86.73%)
LW1-b	3845999	3415733 (88.81%)	1003045 (29.37%)	2412688 (70.63%)
LW2-b	5493010	4264922(77.64%)	473727 (11.11%)	3791195 (88.89%)
LW3-b	3783088	3445658 (91.08%)	814072 (23.63%)	2631586 (76.37%)
LC1-b	4127967	2156247 (52.24%)	784136 (36.37%)	1372111 (63.63%)
LC2-b	3404493	2796269 (82.13%)	896742 (32.07%)	1899527 (67.93%)
LC3-b	4197678	2921439(69.60%)	297191 (10.17%)	2624248 (89.83%)

Total mapped% = Total mapped/ Clean reads; Total mapped: clean reads mapped to the Rfam;

Unique mapped% = Unique mapped/ Total mapped; Unique mapped: reads mapped only one region to the Rfam;

Multiple mapped% = Multiple mapped/ Total mapped; Multiple mapped: reads mapped more than one region to the Rfam.

Supplementary Table S8. Rfam classification.

Type	LW1-a	LW2-a	LW3-a	LC1-a	LC2-a	LC3-a	LW1-b	LW2-b	LW3-b	LC1-b	LC2-b	LC3-b
Cisreg	24925	8156	8709	8363	5693	10192	3525	17830	1734	4657	2659	11165
	0.96%	0.46%	0.49%	0.43%	0.56%	0.86%	0.10%	0.42%	0.05%	0.22%	0.10%	0.38%
lncRNA	10290	3169	4602	3647	2390	4313	925	5316	607	1634	1391	4022
	0.40%	0.18%	0.26%	0.19%	0.23%	0.36%	0.03%	0.12%	0.02%	0.08%	0.05%	0.14%
miRNA	1129235	761621	1070490	1233916	841206	809523	3291679	3679088	3351574	2010638	2679873	2589648
	43.56%	42.67%	60.16%	63.87%	82.36%	68.19%	96.37%	86.26%	97.27%	93.25%	95.84%	88.64%
rRNA	104842	48369	70645	142081	51054	66996	23450	97680	13145	22908	18195	43834
	4.04%	2.71%	3.97%	7.35%	5.00%	5.64%	0.69%	2.29%	0.38%	1.06%	0.65%	1.50%
sRNA	5596	1832	1594	2162	2056	2527	658	7796	280	683	416	2761
	0.22%	0.10%	0.09%	0.11%	0.20%	0.21%	0.02%	0.18%	0.01%	0.03%	0.01%	0.09%
snRNA	162407	62647	109223	86962	28852	57000	48943	169480	29435	50853	45576	86650
	6.26%	3.51%	6.14%	4.50%	2.82%	4.80%	1.43%	3.97%	0.85%	2.36%	1.63%	2.97%
tRNA	1025018	859155	451477	395774	60357	186347	35592	192118	41975	48511	37929	104035
	39.54%	48.13%	25.37%	20.49%	5.91%	15.70%	1.04%	4.50%	1.22%	2.25%	1.36%	3.56%
Others	130107	40060	62538	59077	29822	50177	10961	95614	6908	16363	10230	79324
	5.02%	2.24%	3.51%	3.06%	2.92%	4.23%	0.32%	2.24%	0.20%	0.76%	0.37%	2.72%

The percentage is the ratio of each type in the total types.

All reads which could be aligned to Rfam database were counted by the type of ncRNA in Rfam, the percentage was the ration that the reads of each sample could be matched to the ncRNA compare to the reads of each sample could matched to the Rfam.

Supplementary Table S9. Clean reads mapping to mature miRNAs of pig miRBase.

Sample	Clean reads	Total mapped	Unique mapped	Multiple mapped
LW1-a	8475007	1050909(12.40%)	1027582(97.78%)	23327(2.22%)
LW2-a	6677570	711733(10.66%)	690032(96.95%)	21701(3.05%)
LW3-a	6297075	1015406(16.13%)	992016(97.70%)	23390(2.30%)
LC1-a	11268970	1153757(10.24%)	1113588(96.52%)	40169(3.48%)

LC2-a	2072267	809496(39.06%)	791066(97.72%)	18430(2.28%)
LC3-a	5062403	759375(15.00%)	740546(97.52%)	18829(2.48%)
LW1-b	3845999	1818245(47.28%)	1801218(99.06%)	17027(0.94%)
LW2-b	5493010	3471322(63.20%)	3306188(95.24%)	165134(4.76%)
LW3-b	3783088	2015271(53.27%)	1996037(99.05%)	19234(0.95%)
LC1-b	4127967	1035343(25.08%)	1024272(98.93%)	11071(1.07%)
LC2-b	3404493	1337673(39.29%)	1320364(98.71%)	17309(1.29%)
LC3-b	4197678	2425207(57.77%)	2289918(94.42%)	135289(5.58%)

Total mapped% = Total mapped/ Clean reads; Total mapped: clean reads mapped against pig mature miRNAs of miRBase;

Unique mapped% = Unique mapped/ Total mapped; Unique mapped: clean reads mapped only one region to the pig mature of miRBase;

Multiple mapped% = Multiple mapped/ Total mapped; Multiple mapped: clean reads mapped more than one region to the pig mature of miRBase.

Supplementary Table S10. Reads distribution on the annotated pre-miRNA regions of pig.

Sample	Input reads	Total mapped	Unique mapped	Multiple mapped
LW1-a	7244098	44677(0.60%)	38100(85.28%)	6577(14.72%)
LW2-a	5965837	28840(0.48%)	21235(73.63%)	7605(26.37%)
LW3-a	5281669	39246(0.74%)	32459(82.71%)	6787(17.29%)
LC1-a	10115213	49469(0.49%)	37103(75.00%)	12366(25.00%)
LC2-a	1262771	23983(1.90%)	18545(77.33%)	5438(22.67%)
LC3-a	4303028	30463(0.71%)	23808(78.15%)	6655(21.85%)
LW1-b	2027754	1227738(60.55%)	1194234(97.27%)	33504(2.73%)
LW2-b	2021688	162807(8.05%)	143705(88.27%)	19102(11.73%)
LW3-b	1767817	1142127(64.61%)	1098442(96.18%)	43685(3.82%)
LC1-b	3092624	796683(25.76%)	775313(97.32%)	21370(2.68%)
LC2-b	2066820	1046370(50.63%)	1006762(96.21%)	39608(3.79%)
LC3-b	1772471	113176(6.39%)	99066(87.53%)	14110(12.47%)

Input reads: Clean reads subtract Total mapped in Supplementary Table S9;

Total mapped% = Total mapped/ Input reads; Total mapped: input reads mapped on the annotated pig pre-miRNA regions;

Unique mapped% = Unique mapped/ Total mapped; Unique mapped: input reads mapped only one region to the pig pre-miRNA regions;

Multiple mapped% = Multiple mapped/ Total mapped; Multiple mapped: input reads mapped more than one region to the pig pre-miRNA regions.

Supplementary Table S13. Statistical analyses of expressed mature miRNA and pre-miRNA.

	mature miRNA		pre-miRNA	
Sample	TPM>0	TPM>10	TPM>0	TPM>10
LW1-a	269	209	264	264
LW2-a	268	228	240	240
LW3-a	259	211	237	237
LC1-a	277	232	253	243
LC2-a	250	196	222	222
LC3-a	247	198	245	245
LW1-b	261	177	257	179
LW2-b	288	218	266	253
LW3-b	242	169	247	178
LC1-b	253	181	256	191
LC2-b	257	186	258	190
LC3-b	282	223	260	247

TPM: transcripts per million, TPM(miRNA) = reads(miRNA) /total reads *10^6.

Supplementary Table S14. DE miRNAs in pig breeds

**within-breed comparison**				**inter-breed comparison**			
Large White		Lu Chuan		Mature		Immature	
LW-a vs LW-b Up DE miRNAs (37)	LW-a vs LW-b Down DE miRNAs (17)	LC-a vs LC-b Up DE miRNAs (20)	LC-a vs LC-b Down DE miRNAs (23)	LW-a vs LC-a Up DE miRNAs (2)	LW-a vs LC-a Down DE miRNAs (1)	LW-b vs LC-b Up DE miRNAs (0)	LW-b vs LC-b Down DE miRNAs (1)
miR-1	**let-7f**	miR-1271	**let-7f**	miR-1249	miR-31		miR-34c
miR-101	**miR-10b**	**miR-133a-3p**	**miR-10b**	miR-196a			
miR-1249	**miR-148a-3p**	**miR-143-5p**	miR-124a				
miR-129a	**miR-181a**	**miR-145-5p**	miR-127				
miR-129b	**miR-181d-5p**	**miR-153**	**miR-148a-3p**				
**miR-133a-3p**	**miR-184**	**miR-190a**	miR-149				

miR-133a-5p	**miR-194b-5p**	**miR-205**	**miR-181a**
miR-133b	**miR-199a-5p**	miR-21	**miR-181d-5p**
miR-135	miR-221-3p	**miR-218**	**miR-184**
**miR-143-5p**	**miR-221-5p**	**miR-218-5p**	**miR-194b-5p**
**miR-145-5p**	**miR-301**	miR-22-3p	**miR-199a-5p**
**miR-153**	**miR-361-5p**	**miR-29c**	miR-206
miR-183	**miR-362**	miR-31	miR-214
miR-1839-5p	**miR-370**	**miR-34c**	**miR-221-5p**
**miR-190a**	**miR-376a-5p**	**miR-3613**	miR-27b-5p
miR-190b	**miR-450c-5p**	miR-486	**miR-301**
miR-192	**miR-542-3p**	miR-628	**miR-361-5p**
miR-193a-3p		**miR-9**	**miR-362**
miR-196b		**miR-9-1**	**miR-370**
miR-196b-5p		**miR-9-2**	**miR-376a-5p**
**miR-205**			**miR-450c-3p**
miR-217			**miR-450c-5p**
**miR-218**			**miR-542-3p**
**miR-218-5p**			
miR-218b			
miR-299			
miR-29a			
**miR-29c**			
**miR-34c**			
**miR-3613**			
miR-421-5p			
miR-4336			
miR-493-3p			
miR-582			
**miR-9**			
**miR-9-1**			
**miR-9-2**			

Note: The red markers represent co-expressed up-regulated DE miRNAs and the green markers represent co-expressed down-regulated DE miRNAs in LC and LW pig

Supplementary Table S15. Novel miRNAs

LW-b	LC-b	LW-a	LC-a
NC_010461.4_33692	NC_010461.4_33692	NC_010451.3_32832	NC_010451.3_32832
NC_010461.4_34341	NC_010461.4_34341	NC_010461.4_34341	NC_010461.4_34341
NC_010461.4_34356	NC_010461.4_34356	NC_010461.4_34356	NC_010461.4_34356
NC_010461.4_34336	NC_010461.4_34336	NC_010461.4_34332	NC_010461.4_33692

NC_010461.4_34344	NC_010461.4_34344	NC_010461.4_33692	NC_010461.4_34332
NC_010447.4_21912	NC_010444.3_16017	NC_010461.4_34336	NC_010461.4_34336
NC_010445.3_17465	NC_010451.3_32832	NC_010461.4_34344	NC_010461.4_34344
NC_010461.4_34360	NC_010461.4_34332	NC_010461.4_34354	NC_010445.3_17465
NC_010461.4_34354	NC_010445.3_17465	NC_010461.4_34360	NC_010447.4_21912
NC_010461.4_34366	NC_010461.4_34360	NC_010461.4_34366	NC_010461.4_34354
NC_010461.4_34332	NC_010461.4_34366	NC_010445.3_17465	NC_010461.4_34366
NC_010451.3_32832	NC_010461.4_34354	NC_010450.3_31375	NC_010461.4_34360
NW_003537647.2_34962	NC_010447.4_21912	NC_010444.3_16017	NC_010450.3_31375
NW_003539007.1_35808	NW_003539007.1_35808	NC_010454.3_1921	NC_010454.3_1921
NC_010451.3_33131	NC_010450.3_31375	NC_010447.4_21912	NC_010444.3_16017
NC_010444.3_15468	NC_010444.3_15468	NW_003537647.2_34962	NW_003537647.2_34962
NW_003613308.1_35776	NW_003613308.1_35776	NC_010456.4_6619	NC_010445.3_17469
NC_010456.4_6619	NC_010456.4_6619	NC_010451.3_33131	NC_010451.3_33131
NW_003541201.1_37278	NC_010451.3_33131	NC_010445.3_17741	NC_010456.4_6619
NC_010450.3_31375	NC_010454.3_1921	NC_010445.3_17469	NC_010451.3_33218
NC_010461.4_33630	NW_003541201.1_37278	NW_003539007.1_35812	NC_010458.3_9500
NC_010454.3_1921	NW_003537647.2_34962	NC_010461.4_34362	NC_010461.4_34362
NC_010445.3_17469	NC_010455.4_3901	NC_010447.4_22737	NC_010461.4_33769
NC_010448.3_23529	NC_010461.4_33630	NC_010445.3_18048	NC_010445.3_18048
NC_010458.3_9500	NC_010449.4_26094	NC_010445.3_17585	NC_010458.3_9717
NC_010445.3_18466	NW_003613164.1_35268	NW_003541201.1_37278	NC_010461.4_33630
NC_010453.4_1281	NW_003539007.1_35812	NC_010458.3_9717	NW_003613233.1_35475
NC_010453.4_1283	NC_010445.3_17469	NW_003613233.1_35475	NC_010447.4_22737
NC_010454.3_2163	NC_010453.4_1281	NC_010449.4_26094	NW_003541201.1_37278
NC_010455.4_3927	NC_010453.4_1283	NW_003613164.1_35268	NC_010445.3_18466
NW_003541037.1_37122	NC_010447.4_22737	NC_010458.3_9500	NC_010449.4_26094
NC_010452.3_815	NC_010451.3_33218	NC_010444.3_15340	NW_003613164.1_35268
NC_010452.3_817	NC_010445.3_17741	NC_010445.3_18466	NC_010455.4_4737
NC_010445.3_17585	NC_010445.3_18192	NC_010461.4_33630	NC_010445.3_17741
NC_010455.4_3901	NC_010450.3_31798	NC_010445.3_18465	NC_010444.3_15340
NW_003539007.1_35812	NC_010458.3_9717	NC_010448.3_23529	NC_010455.4_3901
NC_010445.3_18048	NC_010461.4_33705	NC_010461.4_33769	NC_010448.3_24453
NC_010451.3_33218	NW_003613233.1_35475	NC_010461.4_34346	NC_010448.3_23529
NC_010444.3_15340	NC_010461.4_34362	NW_003540104.2_36305	NW_003539007.1_35812
NC_010461.4_34362	NC_010461.4_34346	NC_010444.3_15530	NC_010447.4_22444
NC_010461.4_34346	NC_010445.3_18048	NC_010445.3_17996	NC_010461.4_33447
NC_010451.3_32298	NC_010448.3_23529	NC_010454.3_1960	NW_003613252.1_35533
NC_010451.3_32967	NC_010458.3_9500	NC_010444.3_15468	NC_010444.3_15530
NC_010461.4_33769	NC_010461.4_33699	NC_010446.4_19989	NC_010446.4_19989
NC_010444.3_15530	NC_010461.4_33769	NC_010446.4_19991	NC_010446.4_19991
NC_010445.3_18465	NC_010444.3_15340	NC_010446.4_19995	NC_010446.4_19995
NC_010445.3_18692	NC_010445.3_18465	NC_010447.4_22330	NC_010443.4_14695
NC_010446.4_19989	NC_010445.3_18466	NC_010451.3_32298	NC_010447.4_22330

NC_010446.4_19991	NC_010445.3_18692	NC_010451.3_32967	NC_010454.3_2164
NC_010446.4_19995	NC_010447.4_21208	NC_010461.4_33705	NC_010456.4_7944
NC_010455.4_4477	NC_010455.4_2919	NW_003541037.1_37122	NC_010458.3_9718
NC_010456.4_6570	NC_010455.4_4477	NW_003613073.1_34900	NC_010461.4_34346
NC_010461.4_33638	NC_010456.4_7944	NW_003613308.1_35776	NW_003613073.1_34900
NC_010461.4_33705	NC_010461.4_33638	NC_010444.3_15371	NW_003613233.1_35476
NW_003540104.2_36305	NW_003541037.1_37122	NC_010448.3_25494	NC_010451.3_32298
NW_003613497.1_36815		NC_010455.4_3901	NC_010451.3_32967
		NC_010461.4_33638	NC_010445.3_18465
		NC_010443.4_11897	NC_010447.4_22319
		NC_010447.4_22319	NC_010448.3_23540
		NC_010447.4_22416	NC_010448.3_23542
		NC_010448.3_23540	NW_003539007.1_35808
		NC_010448.3_23542	
		NC_010448.3_25577	
		NC_010455.4_3927	
		NC_010455.4_4477	
		NC_010456.4_6437	
		NC_010456.4_7944	
		NC_010457.4_8785	
		NC_010461.4_33483	
		NC_010461.4_34358	
		NW_003539007.1_35808	
		NC_010447.4_22444	
		NC_010451.3_33218	

TPM: transcripts per million; TPM(miRNA) = reads(miRNA) /total reads *10^6.

Supplementary Table S19. Total number of DE miRNA targets overlap with DEGs. TDETGs: the true differential expression target genes come from the results that the target genes of DE miRNAs and the DEGs overlapped.

DE miRNA sample	Down miRNA target Gene NO.	Up miRNA target Gene NO.	Total TDETGs
within-breed comparison			
LW-a vs LW-b	1612	2029	3641
LC-a vs LC-b	1697	1146	2843
inter-breed comparison			
LW-a vs LC-a	21	71	92
LW-b vs LC-b	37	0	37

Supplementary Table S23. Primers for Real-time quantitative PCR of differential gene and miRNA

gene or miRNA Name	Primer	Primer sequence（5’→3’）	Product Length(bp)	TM(℃)
CCNI	CCNI-F	CAAACCAGAAGTGCCAGTCA	127	60
	CCNI-R	CCCGTCATAGAAGTCATCCA		
SPATA24	SPATA24-F	CCAGAAGCAGCAGGAGAACT	129	60
	SPATA24-R	ATAACGGGAAGGCGACAAC		
KHDRBS3	KHDRBS3-F	CCTACGGACAAGAGGAGTGG	140	60
	KHDRBS3-R	GGACTGGTGGAGAGTGGCTA		
TSGA10	TSGA10-F	TAGGGACGGAGAGGTTTGAA	127	60
	TSGA10-R	GCTCGTTCTGGTGAGTGACA		
GGNBP2	GGNBP2-F	AAGGGAGCAGTAGCAGTGTCA	140	60
	GGNBP2-R	GTCCATCCAACAACCTCTGG		
COL6A2	COL6A2-F	CCCAACTCCCAAGTGTCTGT	131	60
	COL6A2-R	AGCCCAAAGCCAACCAAG		
CD34	CD34-F	CTCCCTCATCTTCCCTCTGA	142	60
	CD34-R	GAGTCCAGTTCCAGCCTCTG		
ssc-RPL32	ssc-RPL32-F	CGGAAGTTTCTGGTACACAATGTAA	96	60
	ssc-RPL32-R	TGGAAGAGACGTTGTGAGCAA		
ssc-miR-301	ssc-miR-301-RT	GTCGTATCCAGTGCAGGGTCCGAGGTATTCGCACTGGATACGACGCTTTG	72	60
	ssc-miR-301-F	GCGGCGGCAGTCCAATAGTATTG		
ssc-miR-194b-5p	ssc-miR-194b-5p-RT	GTCGTATCCAGTGCAGGGTCCGAGGTATTCGCACTGGATACGACTCCACA	74	60
	ssc-miR-194b-5p-F	GCGGCGGTGTAACAGCGACTCC		
ssc-miR-10b	ssc-miR-10b-RT	GTCGTATCCAGTGCAGGGTCCGAGGTATTCGCACTGGATACGACACAAAT	74	60
	ssc-miR-10b-F	GCGGCGGTACCCTGTAGAACCG		
ssc-miR-148a-3p	ssc-miR-148a-3p-RT	GTCGTATCCAGTGCAGGGTCCGAGGTATTCGCACTGGATACGACACAAAG	78	60
	ssc-miR-148a-3p-F	GCGGCGGTCAGTGCACTACAGAAC		
ssc-miR-181d-5p	ssc-miR-181d-5p-RT	GTCGTATCCAGTGCAGGGTCCGAGGTATTCGCACTGGATACGACAACCCA	70	60
	ssc-miR-181d-5p-F	GCGGCGGAACATTCATTGTTGTC		
ssc-miR-181a	ssc-miR-181a-RT	GTCGTATCCAGTGCAGGGTCCGAGGTATTCGCACTGGATACGACAACTCA	74	60
	ssc-miR-181a-F	GCGGCGGAACATTCAACGCTGTC		
ssc-miR-133a-3p	ssc-miR-133a-3p-RT	GTCGTATCCAGTGCAGGGTCCGAGGTATTCGCACTGGATACGACCAGCTG	74	60
	ssc-miR-133a-3p-F	GCGGCGGTTGGTCCCCTTCAAC		
ssc-miR-145-5p	ssc-miR-145-5p-RT	GTCGTATCCAGTGCAGGGTCCGAGGTATTCGCACTGGATACGACAAGGGA	74	60
	ssc-miR-145-5p-F	GCGGCGGGTCCAGTTTTCCCAG		
Reverse primer:	Primer-R	ATCCAGTGCAGGGTCCGAGG		
U6	U6-F	CTCGCTTCGGCAGCACA	106	62
	U6-R/RT	AACGCTTCACGAATTTGCGT		

The following supplementary tables could be seen in the files presented as the excel format.

Supplementary Table S11. Pathways within the breeds for DEGs.

Supplementary Table S12. Pathways between the breeds for DEGs.

Supplementary Table S16. The secondary structure and sequence of the predicted novel miRNAs.

Supplementary Table S17. Biological processes for target genes of DE gene-derived piRNAs.

Supplementary Table S18. Pathway for target genes of DE gene-derived piRNAs.

Supplementary Table S20. Biological processes within the breeds for TDETGs.

Supplementary Table S21. Pathway for TDETGs.

Supplementary Table S22. Biological processes between the breeds for TDETGs.
